# A lysosomal enigma CLN5 and its significance in understanding neuronal ceroid lipofuscinosis

**DOI:** 10.1007/s00018-021-03813-x

**Published:** 2021-04-01

**Authors:** I. Basak, H. E. Wicky, K. O. McDonald, J. B. Xu, J. E. Palmer, H. L. Best, S. Lefrancois, S. Y. Lee, L. Schoderboeck, S. M. Hughes

**Affiliations:** 1 Neurodegenerative and Lysosomal Disease Laboratory, Department of Biochemistry, School of Biomedical Sciences, Brain Health Research Centre, University of Otago, 710 Cumberland Street, Dunedin, 9016 New Zealand; 2 School of Biosciences, Cardiff University, Sir Martin Evans Building, Museum Avenue, Wales, CF10 3AX United Kingdom; 3 Centre INRS-Institut Armand-Frappier, INRS, Laval, H7V 1B7 Canada; 4 Department of Anatomy and Cell Biology, McGill University, Montreal, H3A 2B2 Canada; 5 Department of Biochemistry and Molecular Biology, University of Kansas Medical Center, Kansas City, KS 66160 USA

**Keywords:** Batten disease, Neuronal ceroid lipofuscinosis, Lysosome, CLN5, Neurodegeneration

## Abstract

Neuronal Ceroid Lipofuscinosis (NCL), also known as Batten disease, is an incurable childhood brain disease. The thirteen forms of NCL are caused by mutations in thirteen *CLN* genes. Mutations in one *CLN* gene, *CLN5*, cause variant late-infantile NCL, with an age of onset between 4 and 7 years. The CLN5 protein is ubiquitously expressed in the majority of tissues studied and in the brain, CLN5 shows both neuronal and glial cell expression. Mutations in CLN5 are associated with the accumulation of autofluorescent storage material in lysosomes, the recycling units of the cell, in the brain and peripheral tissues. CLN5 resides in the lysosome and its function is still elusive. Initial studies suggested CLN5 was a transmembrane protein, which was later revealed to be processed into a soluble form. Multiple glycosylation sites have been reported, which may dictate its localisation and function. CLN5 interacts with several CLN proteins, and other lysosomal proteins, making it an important candidate to understand lysosomal biology. The existing knowledge on CLN5 biology stems from studies using several model organisms, including mice, sheep, cattle, dogs, social amoeba and cell cultures. Each model organism has its advantages and limitations, making it crucial to adopt a combinatorial approach, using both human cells and model organisms, to understand CLN5 pathologies and design drug therapies. In this comprehensive review, we have summarised and critiqued existing literature on CLN5 and have discussed the missing pieces of the puzzle that need to be addressed to develop an efficient therapy for CLN5 Batten disease.

## Introduction

Batten disease or Neuronal Ceroid Lipofuscinosis (NCL) is a group of fatal, inherited neurodegenerative disorders that predominantly affect children. Different forms of Batten disease are caused by mutations in thirteen *CLN* genes (*CLN1*-*8* and *10*–*14*), which results in substantial clinical variation, including symptoms and age of onset. The *CLN* genes encode proteins that are found mostly in the endoplasmic reticulum (ER), endosomes or lysosomes. CLN1, 2, 5, 10 and 13 are localised to the lysosomal lumen, CLN3, 7 and 12 are lysosomal membrane proteins, CLN6 and 8 reside in the ER, CLN4 and 14 are cytoplasmic whereas CLN11 is extracellular [1, 2]. CLN1, 2, 10 and 13 act as lysosomal degradative enzymes, CLN5 is proposed to be a lysosomal enzyme, CLN8 and 12 act as transporters, CLN4 acts as a co-chaperone, CLN3, 6 and 11 regulate trafficking of lysosomal enzymes and the functions of the remaining CLNs are unknown [2]). Affected neurons and non-neuronal cells accumulate autofluorescent storage material (ASM), but this deposition appears to be a consequence, rather than a cause, of neuronal dysfunction and death. Research into the roles of *CLN* genes in both neuronal homeostasis and Batten disease is crucial to determine what kills the neurons in disease and how this could be prevented or treated, therapeutically.

Mutations in *CLN5* cause variant late-infantile NCL (vLINCL). The first group to report a Finnish variant of late infantile NCL (fvLINCL) highlighted that the onset of the disease occurred between 4 and 7 years of age [3–5]. Motor deficits, mental deterioration, cognitive impairment, visual impairment and epileptic seizures are the most common presenting symptoms. For vLINCL patients, life expectancy is usually between 10 and 30 years of age [3, 5, 6]. Later studies have reported atypical phenotypes in infantile, juvenile and adult forms of CLN5 Batten disease. In the juvenile and adult forms, symptoms appeared at a later age and the patients survived until the fourth decade of life [7]. In the infantile form, symptoms appeared as early as four months of age, but, to date, is limited to a single reported case [8, 9].

A primary cellular phenotype of all forms of Batten disease is the dysfunction of the cellular waste recycling system—the lysosomes (reviewed in [2]) that are acidic vesicles containing many hydrolytic enzymes. Many of the CLN proteins are localised to the lysosome and their deficits cause lysosomal dysfunction. Among the CLN lysosomal proteins affected in Batten disease (CLN1, 2, 3, 5, 7, 10, 12, 13), CLN5 plays a role in maintaining an acidic environment in the lysosomes, a critical feature for a functional lysosome [10]. However, how CLN5 regulates lysosomal pH, and hence lysosomal homeostasis, is still unclear as the function of the CLN5 protein is unknown. Studies have argued that CLN5 is either a membrane-bound protein or a soluble protein, however, a membrane-bound protein being cleaved and processed into a soluble form appears likely. Several studies have revealed an association between CLN5 and other CLN and non-CLN proteins, indicating that these CLN proteins work together and follow the common cellular pathway. Rare variants of NCL-related genes, such as CLN5 and CLN7, have been described as candidate risk factors for dementia, Alzheimer’s disease and Parkinson’s disease, possibly facilitating the pathogenic mechanisms underlying these diseases [11–13].

Understanding the long-elusive function(s) of CLN5 and how mutations cause Batten disease will not only shed much-needed light on Batten disease mechanisms but will also reveal the significance of lysosomal function in neuronal homeostasis. To date, there is no approved targeted treatment or cure for CLN5 Batten disease. There are more than 150 research and review articles on CLN5, but none of them provide a big picture scenario for CLN5 and where we are heading in terms of future treatment options for CLN5 Batten disease. In this review, our aim is to provide a comprehensive overview of the *CLN5* gene, including its expression and regulation, CLN5 protein expression, processing and post-translational modifications, probable protein functions, and interacting partners, characterisation of the mutations causing CLN5 Batten disease and the use of different animal and cell-based models to study CLN5. We believe that a combined effort to investigate the structure, downstream targets/interactors of CLN5 and the effect of mutations on the transcriptome, epigenome, metabolome, and proteome will help us to understand the underlying pathogenesis and aid the development of mechanism-based treatments.

## CLN5 biology

### The CLN5 gene

In the 1990s, a group of Finnish geneticists mapped the location of the *CLN5* gene to chromosome 13q21.1-q32, using linkage analysis and fluorescence in situ hybridization [14–16]. The Finnish group was the first group who used positional cloning and screening of human fetal brain cDNA library to study CLN5. Their study revealed that *CLN5* cDNA is 4.1 kb long, consisting of four exons with an open reading frame of 1380 bp and a coding sequence of 1221 bp [16, 17]. Other studies from the 1990s reported four exons that span a 13 kb region of genomic DNA. Revised sequences on NCBI Reference Sequence (RefSeq, Accession number: NM_006493.4) show *CLN5* is located on chromosome 13q22 between 76,992,081 bp and 77,005,117 bp. *CLN5* mRNA is 5,243 bp long and the four exons span over a region of 10 kb in genomic DNA. The four exons are 320 bp, 169 bp, 226 bp and 2,060 bp long. There are two reported transcript variants on RefSeq, although the previously reported transcripts encoding 407 aa protein [16] does not currently exist in RefSeq. The present RefSeq CLN5 transcripts consist of a longer transcript encoding a 358 aa protein and a shorter transcript encoding a 197 aa protein. Ensembl reports eight other protein coding transcripts (https://asia.ensembl.org/Homo_sapiens/Gene/Summary?db=core;g=ENSG00000102805;r=13:76990660-77019143), however, the 358 aa protein is considered as ubiquitously expressed. It is not yet experimentally verified whether the expression of other *CLN5* transcripts is dependent on development, tissue type or disease condition. Blast searches for the *CLN5* gene show that there are no known homologous genes or proteins. For *CLN5*, the 407 aa protein has been consistently used in the literature as the reference protein for the past two decades. However, due to the change of the protein length to 358 aa in the current RefSeq database, in this review, we have adapted to the new CLN5 358 aa protein and modified the mutation locations accordingly. For the remainder part of this review, we will refer to the 358 aa CLN5 protein, rather than the previously referenced 407 aa protein.

Often 5′ and 3′ flanking genes play roles in gene expression regulation [18]. Using traditional and computational methods for transcript identification, Klockars et al*.* [17] found that the CLN5 transcript is flanked by four more genes, namely *cDNA761, Ribosomal protein L7 pseudogene, RNAse helicase A/nuclear DNA helicase II / leukophysin pseudogene and PAM.* However, the recent RefSeq database shows the locations of these four genes to be on different chromosomes. According to the UCSC genome assembly, the CLN5 adjacent genes are ubiquitin ligase complex subunit *FBXL3*, E3 ubiquitin ligase *MYCBP2* and pseudogenes *DHX9P1* and *RPL7P44*. Their roles in relation to CLN5 function has never been explored. Hence it is still unknown if these or other genes are positionally important for the regulation of CLN5 gene expression.

### Regulation of CLN5 expression

Savukoski et al*.* [16] suggested three possible 50 bp promoter sequences, but since then, no other study has defined the promoter region of *CLN5*. It remains unclear what regulates *CLN5*, with only a few studies and prediction analyses investigating *CLN5* regulation. GeneHancer predicts 38 promoter/enhancer elements regulating the *CLN5* gene [19]. There are 58 predicted CpG islands near the *CLN5* transcription start site (source: UCSC GRCh38/hg38 assembly), but no known experimentally verified methylation or acetylation data are available in the literature. The first study to suggest a transcription factor associated with CLN5 [20] used somatic cell hybrid analysis to show that the Brn-3A transcription factor *POU4F1* lies within the critical region defining the *CLN5* locus. However, since the 1996 study, no other researchers have verified this transcription factor, raising questions about the actual association between *POU4F1* and *CLN5*.

While investigating the promoters of 96 genes encoding known lysosomal proteins, Sardiello et al. [21] revealed the Coordinated Lysosomal Expression and Regulation (CLEAR) gene network. A ChIP-Seq analysis from the study found that Transcription Factor EB (TFEB) binds to the CLEAR motif. This motif was enriched in the promoters of 68 out of 96 genes, including *CLN5* and other genes regulating lysosomal function and biogenesis. Upon manipulation of TFEB expression in HeLa and HEK293 cells, lysosomal gene expressions were altered–including that of *CLN1*, *CLN2*, *CLN3*, *CLN5*, Cathepsin D (*CLN10*) and Cathepsin F (*CLN13*). For *CLN5*, the binding sites reported by Sardiello et al*.* were mapped to + 50 (CTCAAGTGTG) and + 74 (TTCAGGTGCC) on the promoter of *CLN5* [21]. A 2011 study by Palmieri et al. confirmed the regulation of *CLN* genes via TFEB using microarray analysis, deep sequencing of chromatin immunoprecipitate, unbiased genomic and expression meta-analyses in HeLa cells [22]. However, apart from mapping the TFEB binding site on the *CLN5* promoter, there is no experimental validation data available showing TFEB to be the sole regulator of *CLN5*. Furthermore, the experiments in the aforementioned studies were performed in non-neuronal cells. Neuronal cells, with their unique morphology, are expected to harbour additional transcription regulatory elements when compared with HeLa and HEK293 [23]. TFEB is negatively regulated by the mechanistic target of rapamycin complex 1 (mTORC1), which disrupts the nuclear translocation of TFEB and inhibits autophagy [24]. Additionally, TFEB is also negatively regulated by serine/threonine kinase Akt (protein kinase B,) and blocking Akt using trehalose in *Cln3*^*Δex7−8*^ mice showed improved survival [25]. Sheep and mouse models of CLN5 Batten disease also show autophagy impairment [10, 26]. Whether a similar improvement of disease phenotype in CLN5 Batten disease is possible by enhancing TFEB-mediated lysosomal gene activation needs further investigation.

Currently, there is no knowledge of other gene regulatory elements, such as non-coding RNAs, for *CLN5*. MicroRNAs (miRs) and long non-coding RNAs are the most well-known non-coding gene regulatory elements. miRWalk, a miR prediction tool that provides both predicted and validated miRNAs targets, showed miR-124-3p (a brain-specific miR) has been validated to target *CLN5* [27]. On further investigation into the literature and its associated supplementary information, it became evident that miR-124-3p has been mistakenly stated as a *CLN5* regulator. The same study involved a second miR, miR-1 (not a brain-specific miR), which showed possible regulation of *CLN5*, but further experimental validation is required. Interestingly, miR-1 is upregulated in retinitis pigmentosa, a group of inherited retinal degenerative diseases [28], and is of particular interest given vision loss and retinal degeneration are well-known clinical signs in CLN5 Batten disease [3, 5, 26]. Furthermore, several other miR prediction tools predicted miR-138-5p to target *CLN5*. Although miR-138 is regarded as a potential molecular regulator of human memory function [29], without further experimental validation, it would be futile to extrapolate if miR-138-mediated moderation of *CLN5* contributes to impaired brain function in CLN5 Batten disease.

### CLN5 translation

There are four ATG sites and potential start codons at the 5′ end of the *CLN5* gene, which could lead to four forms of the CLN5 protein. The four respective methionines are at positions 1, 30, 50 and 62 aa, which when translated would produce polypeptides of 407, 378, 358 and 346 aa. Based on the consensus analysis, the first ATG was considered the site of translation initiation resulting in a predicted polypeptide of 407 aa weighing ~ 46 kDa [16, 17, 30, 31]. A recent update to the RefSeq sequence omitted the initial 49 aa, resulting in a 358 aa protein, corresponding to the use of the third ATG. Comparison between the new 358 aa and the old 407 aa CLN5 sequences suggests that the new sequence only has two methionine sites at the first aa (previously 50th aa) and the twelfth aa (previously 62nd aa).

Although cell-free analyses of CLN5 showed the protein to use alternate start sites, in-cell analyses point towards a single start site. In 2002, two studies investigated the potential use of alternative start codons in CLN5. Vesa et al*.* [32] performed a cell-free translation assay resulting in four forms of CLN5 with molecular weights 47, 44, 41 and 39 kDa. Upon mutation of the methionine sites, individually as well as together, in fibroblast-like COS-1 cells, the study showed that the COS-1 translation machinery is able to use at least one methionine located at 1, 30 or 50 aa, however, their antibody failed to detect the 39 kDa band. Surprisingly, the study ignored higher molecular weight species (> 50 kDa), which were shown to be glycosylated forms of CLN5 in later studies. Isosomppi et al*.* [30] performed similar cell-free translation assays and confirmed the aforementioned CLN5 molecular weights [30]. In the same study, when the group used a kidney cell line BHK-1, they found CLN5 being expressed as a 60 kDa glycoprotein, which when deglycosylated appeared as a 38 kDa protein. However, the specificity of the antibody used in the study by Isopompi et al. is questionable, as multiple bands were observed in both cellular and media extracts, especially after deglycosylation. The 38 kDa CLN5 protein would be the product of the methionine at 50 aa position, which is the start site on the currently favoured 358 aa sequence. The difference of CLN5 product size might suggest that expression and posttranslational modifications of CLN5 varies between cell lines and perhaps have tissue-specificity or overexpression of CLN5 alters glycosylation pattern. Our unpublished data in a kidney cell line, HEK293FT, and human-induced pluripotent stem cells (iPSCs) have shown a predominant glycosylated CLN5 form at ~ 60 kDa, which when deglycosylated, generates a ~ 38 kDa CLN5 species (J. Palmer, Basak, Hughes, unpublished).

### Tissue expression of CLN5

Tissue expression of the CLN5 transcript and protein has been thoroughly studied in the last three decades and we have summarised the data (Table 1). The first comprehensive CLN5 tissue expression study was performed by Savukoski et al. [16], where the authors showed the highest level of adult *CLN5* expression in the aorta, kidney, lung and pancreas (Table 1). In the fetus, the highest signal was obtained in the thymus as compared to the brain, and other peripheral organs, all of which showed uniform expression [16] (Table 1). GTeX portal data indicate that the highest expression of *CLN5* is in the thyroid followed by the tibial nerve, ovary, tibial artery, coronary artery and aorta, with brain tissue showing moderate expression (Table 1). Heinonen et al*.* [33] showed that CLN5 had weaker expression in human embryos, as compared to that of CLN1, at the beginning of cortical neurogenesis, and its subsequent expression increased with cortical development. Later, Holmberg et al. [34] used mouse tissue to show that *Cln5* is expressed in the brain and peripheral organs (Table 1), where some tissue showed expression of more than one *Cln5* transcript. The different *Cln5* transcript expressions could suggest different isoforms or differential processing of *Cln5* in different tissues [34]. Cln5 protein expression in these organs was also confirmed by De Silva et al. [35] (Table 1).

**Table 1:** CLN5 expression in human and mouse tissues

References	Method used	Human CLN5 expression	Murine CLN5 expression
[16]	Northern blot, RNA hybridisation	Adult: High in aorta, kidney, lung, pancreas Foetus: High in thymus; uniform in brain, heart, kidney, liver spleen, lung	–
GTeX portal	RNA sequencing, microarray	Highest to lowest: Human thyroid, tibial nerve, ovary, tibial artery, coronary artery, aorta, brain	–
[33]	In situ hybridisation, immunohistochemistry	*CLN5* expression increases with cortical development E37: expressed in ventricular zone and peripheral cells, E76: expressed in cells leaving ventricular zone and migrating towards cortical regions	–
[34]	Northern blot	–	Highest to lowest: liver, kidney, skeletal muscle, lung, heart, spleen, brain, testis
[35]	Western blot	–	Highest to lowest: liver, kidney, rectum, spleen, jejunum, colon, heart, lung, adipocyte, testis, muscle, brain, thymus
[34, 36, 37]	Northern blot, in situ hybridisation, immunohistochemistry, polymerase chain reaction	–	*Cln5* expression increased with development Particularly abundant expression in cerebellar Purkinje cells, cortical neurons, hippocampal pyramidal cells, hippocampal interneurons, glial cells, neurons in white matter and brain stem, hypothalamus, retrosplenial granular cortex, paraventricular thalamus, ventricular regions, choroid plexus

In the brain, throughout human embryonic development, CLN5 protein is found in the ventricular zone and in some more peripheral cells at E37. At E76, CLN5 is expressed in cells leaving the ventricular zone and migrating toward the cortical region (Table 1). In the cortical plate, CLN5 is mostly seen perinuclear, and CLN5-negative cells are interspersed among strongly positive cells [33]. In mice, Cln5 is expressed from E15 and steadily increases from then on. At P7, 14, 24, and 60, Cln5 strongly stained cerebellum, cerebral cortex and hippocampus (Table 1). These areas have been noted to particularly degenerate in CLN5 patients’ brains. Cln5 is also found in the hypothalamus. In the hippocampus, CA3 pyramidal cells are specifically labelled, while the labelling in CA1 was weaker. In large neurons, a granular appearance existed predominantly in the cell soma with some staining extending into neurites. The Cln5 protein localised to neuronal extensions did not co-localise with Lamp1, which could indicate an extra-lysosomal function in neurons [34].

More recently, Cln5 has been found in the mitotically active zone of the cerebellum at E18.5, in the inner migratory zone of the external granule layer, and in neurons located in the white matter and brain stem, including the facial nucleus [36] (Table 1). High levels of Cln5 were observed in the pons region across the cerebellum. During postnatal development up to day P7, Cln5 was found in the external granular cell layer of the cerebellum and on P7 the Purkinje cell layer showed strong expression (Table 1). Expression in the internal granule layer of the cerebellum lasted into adulthood. During postnatal brain development, expression in the hippocampus remained strong: from P1 in pyramidal cells in CA2 and CA3, but also the dentate gyrus. This pattern continued into adulthood. Large neurons in the hippocampal region and cerebral cortex as well as cortical neurons were positive (Table 1). The following regions also stained positive throughout development: old cortex, retrosplenial granular cortex, hypothalamus, paraventricular thalamus, ventricular regions, choroid plexus [36] (Table 1).

Holmberg et al. [34] confirmed that mouse brain tissue showed increased *Cln5* expression with development, a finding later supported by Schmiedt et al. [37] and Fabritius et al. [36] (Table 1). Another interesting observation made by Savchenko et al. [38] confirmed that Cln5 loss caused impaired neurogenesis. All of these data suggest that Cln5 plays a crucial role in brain development. Cln5 expression is concentrated mostly in the cerebral cortex and cerebellum in both developing and adult mouse brain tissue [34] (Table 1). Immunohistochemical staining of Cln5 in mouse brain tissue confirmed immunoreactivity in neurons and glia [34] (Table 1). Interestingly, a later study by Schmiedt et al. [37] showed *Cln5* mRNA expression is highest in microglia, followed by astrocytes, oligodendrocytes and neurons, although it is not clear if these measurements were done in developing or adult mouse brain. By inducing microglial activation using lipopolysaccharide, Schmiedt et al. [37] showed that Cln5 expression increased threefold in microglia, suggesting that Cln5 might also have a role to play in the immune response.

Glial cells have more than a supporting role to play in the brain. Major neurodegenerative diseases have confirmed pathologies in the glial cells that lead to amplified neurodegeneration [39–41]. Likewise, impaired glial morphology and function have been shown in Batten disease, as highlighted by Parviainen et al. [42] and Lange et al. [43] in Cln3 and Cln1 deficient mice, respectively. These studies also showed that defective mouse glial cells could kill healthy cortical neurons, suggesting glial activation as an indication of neurodegeneration in Batten disease. Furthermore, microglial activation and astrocytosis have been shown in CLN5 Batten disease patients, especially in regions where neuronal demise was more prominent [44]. Cln5 knockout (^−/−^) mice show enhanced microglial activation and impaired myelination of oligodendrocytes [37]. Hence, the higher expression of CLN5 in glial cells suggests its regulatory role of glial cell function and a protective role in neurons. CLN5 disease patients often present loss of myelin [37, 45], and genes related to myelination are downregulated in *Cln5*^*−/−*^ mouse brains [46, 47]. Although the loss of myelination is not considered as the primary effect of CLN5 loss, defects in myelination are not only limited to humans but also have been reported in mouse models, which eventually could lead to axonal loss and subsequent neuronal loss. Defects in myelination could lead to axonal loss and subsequent neuronal loss. The volume of two major white matter tracts showed no significant atrophy at one and three months in *Cln5*^*−/−*^ mice. Cultures of oligodendrocytes from *Cln5*^*−/−*^ mice showed that fewer of them differentiated from progenitor cells. They retained small soma and numerous highly branched processes [34, 35, 37, 48].

The drastic neurological symptoms of all forms of Batten disease tend to overshadow any peripheral symptoms. However, the near-ubiquitous expression of CLN5 is a key observation suggesting that CLN5 possesses functions beyond the brain. Being a lysosomal protein, the absence or mutation of CLN5 is expected to affect all cell types. It is well-known that progressive development of cardiac pathology in CLN3 Batten disease is associated with the progression of the disease [49]. Furthermore, Australian cattle dogs with *CLN5* mutation show deposition of autofluorescent inclusions in the cardiac muscle of the heart ventricle [50]. It is expected that such peripheral symptoms will continue to emerge in the years to come. The higher expression of CLN5 in peripheral tissues as compared to the brain and these non-CNS symptoms will have implications when it comes to maximising therapeutic efficacy, which are currently developed with a predominant focus on the CNS. Hence, in future in vivo studies involving CLN5, assessment of extraneuronal pathologies should be intensified.

### CLN5 post-translational modifications, processing, trafficking and protein interactions

#### Proteolytic processing

**Fig. 1:**
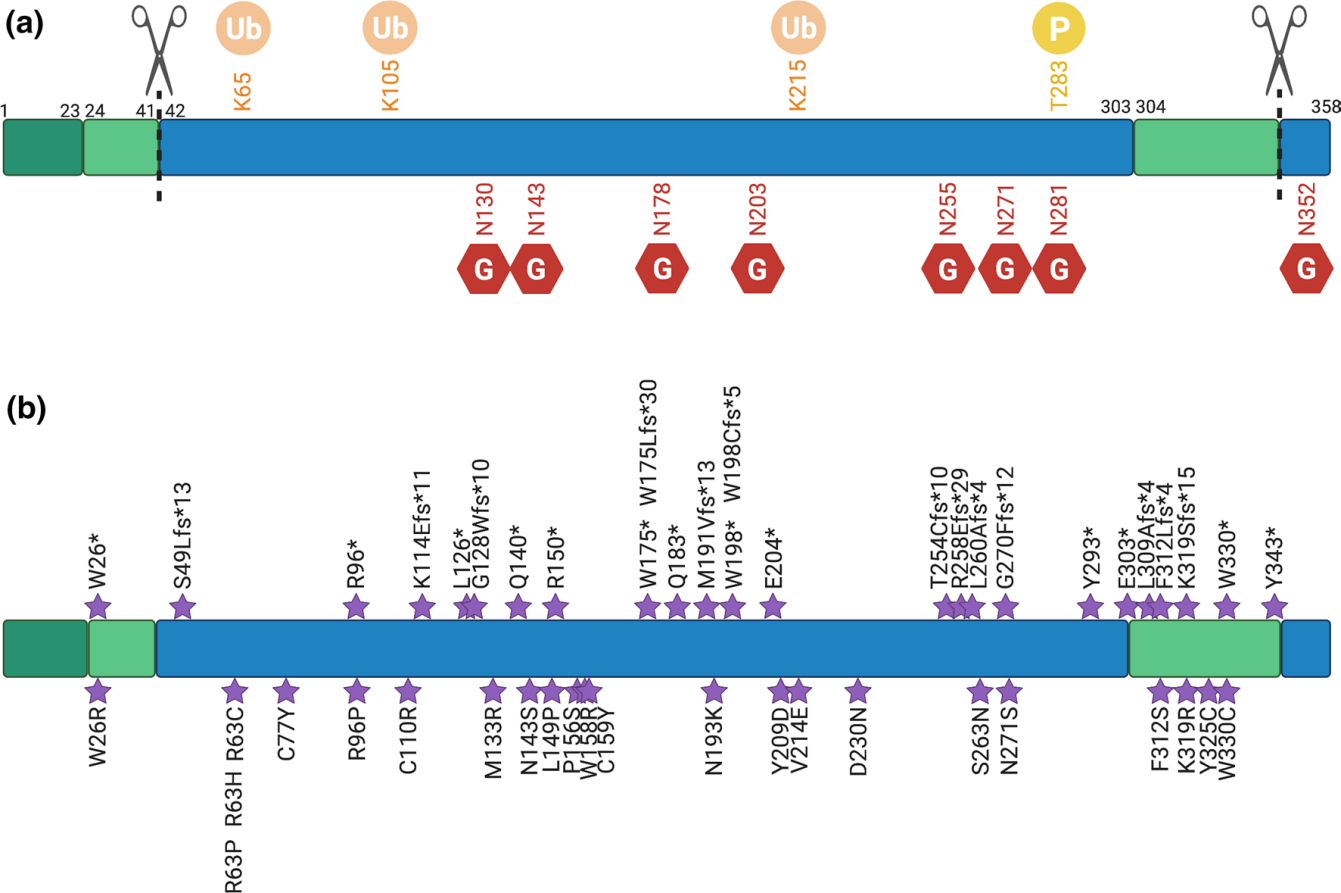
CLN5 is a soluble lysosomal glycoprotein. a: The CLN5 protein contains a signal peptide (dark green), two predicted transmembrane regions (pale green), and a large soluble CLN5 domain within the lysosomal lumen (blue). The signal peptide is cleaved to form the mature protein, and there is also a cleavage site near the C-terminus. Eight experimentally validated N-linked glycosylation sites (red) are essential for the intracellular trafficking of CLN5 and there are also three predicted ubiquitylation sites (orange) and one predicted phosphorylation site (yellow). **b:** Disease-causing mutations (purple) are found throughout the CLN5 protein. Truncating and frameshift mutations are shown on the upper row and amino acid substitutions on the lower row. Created with BioRender.com

Human CLN5 is synthesised as a preproprotein CLN5 nascent polypeptide. The precursor protein moves into the lumen of the endoplasmic reticulum (ER) where the N-terminal signal peptide is cleaved [35, 51–53] (Fig. 1a) and oligosaccharide side chains (known as glycans) are added to the polypeptide, all of which happens co-translationally. More recently, C-terminal cleavage of CLN5 has been suggested to occur in an acidic environment [35] (Fig. 1a). Lysosomal targeting of CLN5 is not dependent on the starting methionine [51].

CLN5 was originally predicted to be an ER transmembrane protein [16] and early data supported this hypothesis, with CLN5 showing at least one transmembrane domain [32]. Endogenous CLN5 polypeptides detected from human fibroblasts were reported to be membrane-bound [54]. Larkin and co-workers investigated the membrane association of CLN5 in more detail in HEK293 and HeLa cells [52]. In this comprehensive study, Larkin et al. tagged CLN5 with human influenza hemagglutinin (HA) in different positions and detected several CLN5 bands on a western blot: 73 kDa glycosylated full-length uncleaved precursor, 60 kDa glycosylated and cleaved, 50 kDa unglycosylated full-length uncleaved precursor, 35 kDa unglycosylated and cleaved and a 15 kDa signal peptide fragment [52] (Table 2). In a pulse-chase experiment, the 60 kDa mature form of CLN5 present after a 50 min pulse indicated that N-glycosylation and signal peptide cleavage occur co-translationally [52]. This was in congruence with an earlier study by Schmiedt et al., where the authors found a 60 kDa proform and a 50 kDa mature human CLN5 detected with a C-terminal antibody in HeLa cells [51] (Table 2). A protease protection assay in the study conducted by Larkin et al. [52] indicated that the N-terminal tail was located in the cytoplasm and the C-terminus in the ER lumen. Subcellular fractionation of cells expressing various tagged CLN5 proteins resulted in the N-terminally tagged version being mainly found in the membrane fraction – strengthening the argument for an N-terminal transmembrane domain. However, CLN5 with a C-terminal tag and a tag after the signal peptide cleavage site were also identified as membrane-associated. Mature CLN5 remained tightly associated with the membrane but did not behave like a typical integral membrane protein [52].

**Table 2:** Studies showing different CLN5 processing in different species and CLN and non-CLN protein interactors

CLN5 in different species	UniProt ID	Length (aa)	Precursor	Mature	De-glycosylated (kDa)	Signal-peptide (kDa)	Interactions
hCLN5	O75503	358	60 kDa [30]	–	EndoH: 40 kDa	–	CLN1, CLN2, CLN3, CLN6, CLN7, CLN8 [32, 47, 70–72, 155] Rab7, Rab5, CerS, Vimentin, H2AFZ / H3F3A / H1H4H / H2A type 2-C [75–77] FBXo6, OBP2A, CALR3, LIPH [80–82, 84, 85]
			73 kDa glycosylated and uncleaved [52]	60 kDa glycosylated and cleaved [52]	EndoH: 50 kDa, unglycosylated full length uncleaved precursor, 35 kDa unglycosylated and cleaved [52]	15 kDa [52]	
			Preproprotein from which N-terminal signal peptide is cleaved co-translationally [51]	60 kDa proform, reduced upon block of intracellular transport (ER to Golgi) 50 kDa mature form, increased upon block of intracellular transport (ER to Golgi) [51]	–	–	
			PreproCLN5: uncleaved unglycosylated at 50 kDa ProCLN5: uncleaved, glycosylated: slightly above 75 kDa [53]	Slightly above 50 kDa: cleaved and glycosylated mature CLN5 [53]	EndoH and PNGase F: preproCLN5 not affected ProCLN5 converted to preproCLN5 [53]	Slightly below 20 kDa [53]	
			56 kDa proprotein [35]	52 kDa (C-terminally cleaved) [35]	PNGaseF: Proprotein and mature protein still discriminable [35]	–	
mCln5	Q3UMW8	341	48 + 50 kDa [34]		PNGaseF:34 kDa. EndoH: 35 kDa In vitro translated: 37 kDa and 3 kDa SP [34]	–	–
			Up to 75 kDa smear + 47 kDa band [32]		EndoH: High molecular smear gone, 2 bands 2 kDa apart remained PNGaseF: Only 47 kDa band remained [32]		
oCLN5	A2TJ54	361		60 kDa [1]	EndoH: 38 kDa [1]	–	–
				60 kDa [Xu and Hughes, unpublished]	EndoH: 46, 42 and 38 kDa [Xu and Hughes, unpublished]		

The studies by Isosomppi et al. [30] and Holmberg et al. [34] showed the presence of glycosylated CLN5 polypeptides in the media of cultured cells and human CLN5 and mouse Cln5 found in the soluble fraction. Isosomppi et al. suggested that an N-terminal signal peptide would be cleaved like other soluble lysosomal proteins [30]. Evidence supporting this hypothesis included staining in Golgi [32] and exclusive ER localisation with an N-terminal tag [52]. In a 2017 study [53] a topology assay performed in perforated intact HeLa cells showed that the N-terminal tail of CLN5 was digested, placing it in the cytosol. Mature CLN5 was protected and therefore appears to be a soluble protein within the lumen of organelles. This supports a model with preproCLN5 (unglycosylated) and proCLN5 (glycosylated) as type II transmembrane proteins cleaved into a mature soluble protein. A further membrane separation assay focused on the ER, finding the majority of HA-tagged CLN5 to be associated with the membrane fraction and a small amount in the soluble fraction. This supports the topology assay data, by placing preproCLN5 and proCLN5 as membrane-associated protein [53]. CLN5-HA was found in the membrane fraction when the internal membranes were left intact. When the membranes were isolated, CLN5 and other lysosomal proteins are released into the soluble fraction [53], contradicting the membrane-associated results shown by Larkin et al. [52].

In 2015, De Silva et al. [35] studied the endogenous expression of CLN5 in HEK293 and HeLa cell lines, which resulted in two specific major bands on western blots: one around 56 kDa, and another around 52 kDa, indicating a proprotein and a different mature form (Table 2). Using an over-expressed myc-tagged protein, and a C-terminal antibody, the authors revealed that the C-terminus of CLN5 is cleaved. N352Q, a CLN5 mutant that deletes a glycosylation site, accumulated in the Golgi network and in the media [55], as both proprotein and mature variants. This suggested that the proteolytic processing can occur in the Golgi network which is mildly acidic. Secretory vesicles from the trans-Golgi network (TGN) also contained proprotein of overexpressed CLN5, but this secreted proprotein was larger (> 10 kDa difference) and the authors suggested further modification such as fucosylation and sialylation when being transported from TGN to outside of the cells [35]. Interestingly, overexpressed sheep CLN5 with a myc-tag at the C-terminus was also only detectable in pre-lysosomal compartments, ER and Golgi, and also secreted [1] supporting C-terminal cleavage prior to lysosomal localisation.

While Schmiedt et al. [51] pointed towards cleavage of the signal peptide at residue 96 aa (New RefSeq sequence–47 aa), mass spectrometry after immunoprecipitation identified CLN5 fragments beginning from residue 93 (New RefSeq sequence–44 aa), indicating a cleavage site at 92nd aa (New RefSeq sequence–43rd aa). There is some ambiguity that the mature protein could either be residues 93–407 aa or 89–407 aa [53] (New RefSeq sequence–44–358 aa or 40–358 aa). Jules and colleagues discovered that the specific enzyme cleaving the CLN5 signal peptide is SPPL3. The SPP/SPPL family are known to cleave type II transmembrane proteins at different intracellular locations [56] (Friedmann 2006). Inhibition of one of the members, SPPL3, which localises to the ER and Golgi apparatus, prevented the cleavage of CLN5 from proCLN5 [53]. Transfection with a catalytically inactive form of SPPL3, as well as knockdown of SPPL3 resulted in proCLN5 accumulation [53]. While classical signal sequences are usually 15–20 aa long [57] and cleaved during translation, CLN5 cleavage likely occurs after insertion into the ER membrane. In an experiment where the N-terminus was replaced by a classical signal peptide of a lysosomal enzyme, CLN5 was still correctly localised to lysosomes in HeLa cells, indicating that its N-terminus is not critical for its localisation. Further experiments indicated an additional cleavage event via SPPL2b on the already cleaved N-terminal signal peptide. This intracellular domain is rapidly degraded by the proteasome [53].

#### Post-translational modifications of CLN5

Glycosylation of CLN5 renders it a soluble and more stable protein undergoing a series of post-translational modifications for proper folding and trafficking [32, 51]. Localisation studies have suggested that various N-glycosylation sites of CLN5 have different effects on folding, trafficking and the lysosomal function of CLN5 [55]. The CLN5 sequence of 358 aa with a calculated molecular weight of 48.36 kDa [16, 55], http://www.rcsb.org/structure/6R99) harbours 8 potential N-glycosylation sites: three encoded in exon 3: N130, 143, 178 and five encoded in exon 4: N203, 255, 271, 281 and 352 (Fig. 1a). The utilisation of these sites was confirmed by multiple studies using the enzymes Endoglycosidase H and Peptide N-glycosidase F [30, 32, 55, 58]. Point mutants eliminating individual glycosylation sites resulted in an approximately 2.5 kDa reduction. There were, however, slight mobility differences between them indicating that the modifications are not identical[55]. A patient mutation D230N introduces an extra potential glycosylation site and indeed leads to an increase in molecular weight of about 2.5 kDa [55].

Five N-glycosylation residues on human CLN5, N130, 203, 255, 271 and 281 (Fig. 1a), are essential for proper protein folding [30, 55, 59]. In HeLa cells, mutations in N130, 203, 255 or 271 (Fig. 1a, b) lead to mis-localisation of CLN5 to the ER. Mutation of N281 resulted in CLN5 localisation to both lysosomes and ER. The other two residues, N143 and N178, do not appear to play roles in CLN5 folding or trafficking but were suggested to be crucial for the functionality of the CLN5 protein in lysosomes [55]. The human CLN5 N352 glycosylation site, not present in mouse, rat or zebrafish proteins [55], is essential for the lysosomal localisation of human CLN5. Without it, CLN5 accumulates in the Golgi membranes temporarily and is then secreted into the media. The mannose 6 phosphate (M6P) modification on N352 is the major determinant marking human CLN5 to the M6P receptor (MPR)-dependent lysosomal trafficking pathway [55, 60, 61]. Even though N271 and 281 of human CLN5 were identified as containing M6P moieties, they are not determinants for MPR-dependent transport for CLN5.

Altered posttranslational processing of N271 has recently gained more attention when it was identified as a risk variant for Alzheimer’s disease [11]. As reported by Moharir et al. [55], this variant leads to ER entrapment of CLN5 and decreased amounts in the media of cultured cells. Overexpression of the N271S mutant in HeLa and N2a cells also resulted in a small decrease in intracellular full-length amyloid precursor protein and an increase in the ratio of procathepsin D to mature cathepsin D [11].

While N-glycosylation of CLN5 is well established, other posttranslational modifications have not been studied in great detail so far. High throughput studies however give some insight into CLN5 post-translational modifications. Phosphosite.org (https://www.phosphosite.org/proteinAction.action?id=3641403&showAllSites=true, accessed 8/5/2020) lists three ubiquitylation sites: K65 [62], K105 [62–64] and K215 [65], as well as one phosphorylation site on T283 [66] (Fig. 1a), but these sites remain unverified experimentally, and their function remains to be established.

#### CLN5 localisation and trafficking

**Fig. 2:**
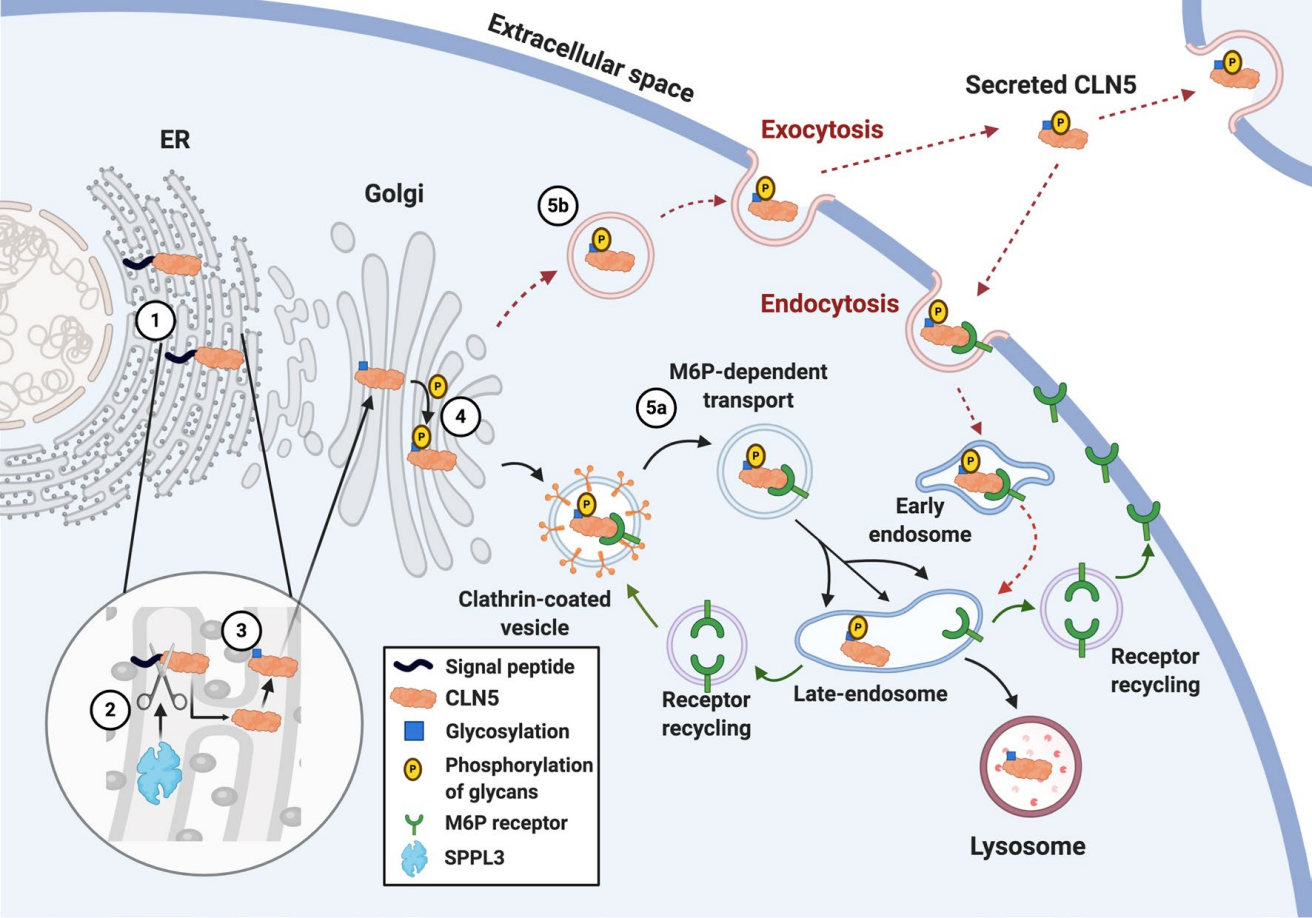
CLN5 is processed in the ER and trafficked to the lysosomes via M6P-dependent pathways. CLN5 is synthesized as a preproprotein (1). The signal peptide is cleaved from the pre-proform of CLN5 in the ER by SPPL3 (2) and CLN5 gets glycosylated (3). CLN5 is transported to the Golgi apparatus where complex-type sugars are added and some mannose residues are phosphorylated at carbon 6 (4). CLN5 is then transported through the early endosome and trafficked to lysosomes via the M6P-dependent pathway (indicated by solid black arrows) (5a). A small amount of CLN5 protein also joins the secretory pathway (indicated by dashed red arrows) and is secreted into the media via exocytosis (5b). CLN5 can re-enter the cell via M6P receptor-mediated endocytosis and transported to the early and late endosomes and finally to the lysosomes (indicated by dashed black arrows). Created with BioRender.com

The subcellular localisation of CLN5 is recognised as lysosomal (Fig. 2), as well as some co-localisation with Golgi- and endoplasmic reticulum (ER) markers which was subsequently lost upon block of protein synthesis [30, 32]. When CLN5 was tagged at different locations, either at the N-terminus or after the signal peptide cleavage site, the tagged forms localised in a similar manner to the untagged CLN5 [52]. After a 1-hour chase in a pulse-chase experiment, both tagged and untagged CLN5 were detected in the extracellular media [52].

Glycosylated human CLN5 is suggested to be trafficked via the M6P-dependent pathway (Fig. 2) [60, 61, 67]. Phosphorylation of CLN5 glycans followed by binding to MPR (Fig. 2), leads to sorting and movement of CLN5 to the late endosome where the MPR disassociates as the pH reduces from 6.5 to 5.5. Next, the glycans are dephosphorylated and CLN5 is transported to lysosomes, while the MPRs are recycled to the TGN or plasma membrane (Fig. 2) [68]. In addition to the M6P-dependent pathway, CLN5 also uses an alternative route to lysosomes [51] (Fig. 2). A fraction of M6P-phosphorylated CLN5 proteins “miss” binding to the intracellular MPR in the Golgi apparatus, and is instead enclosed into secreted protein budding vesicles, and secreted into the media via exocytosis (Fig. 2). These secreted CLN5 molecules can be taken back up into the cells by binding to plasma membrane MPR, transported to the early and late endosomes, and finally to lysosomes (Fig. 2) [69]. In support of the unconventional route of CLN5 secretion, Huber et al. showed that in *Dictyostelium*, CLN5 secretion during the early stage of *Dictyostelium* development occurred by bypassing the Golgi complex [58].

#### Protein–protein interactions

With the CLN5 structure solved and the study predicting it to be a lysosomal protease (http://www.rcsb.org/structure/6R99), it will be interesting to discover the true substrates of CLN5. So far, several CLN and non-CLN candidate proteins have been shown to interact directly or indirectly with CLN5, which is summarised in the following sections.

Like CLN5, several other NCL proteins are trafficked through the ER–Golgi network and localised in the lysosomes, suggesting that they may share common interlinked pathways, especially when mutations in all CLN genes show similar pathologies [70–72]. CLN5 interacts with several other NCL proteins in vitro using human or murine overexpression systems, including PPT1/CLN1, TPP1/CLN2, CLN3, CLN6 and CLN8 [32, 47, 70–72] (Table 2).

CLN5 and CLN1/PPT1 colocalise from the ER to the lysosome [47, 70] CLN5 is thought to already interact with premature ER forms of CLN1 which are di- or triglycosylated [70]. In CLN5 deficient patient fibroblasts, overexpression of a trafficking-deficient variant of CLN5 that was retained in the ER also restricted CLN1 to the ER. Overexpression of CLN1 in cell culture restored localisation of otherwise ER-retained mutated CLN5 (Finnish major mutation, discussed later) to lysosomes [70]. However, overexpression of CLN5 could not rescue a localisation-defective mutant of CLN1 despite the interaction between the two proteins [70], suggesting the significance of CLN1-CLN5 interaction in maintaining CLN5 localisation and function. Interactions between CLN5 and mature CLN2 are reported to occur after the proteins have left the ER[47], possibly in late endosomes or lysosomes [70]. Mutations (Finnish major mutation, Swedish and European mutations) in CLN5 resulted in the loss of interaction with CLN2. The CLN2 activity in CLN5 patient’s fibroblasts is increased [32]. Trafficking of CLN3 is partially affected by the simultaneous expression of the aforementioned mutated CLN5, possibly suggesting that CLN5 and CLN3 proteins interact in the ER [32, 47]. Studies have suggested that CLN5 and CLN6 are likely to interact in the CNS [70, 73]. Despite different cellular localisations of mature lysosomal CLN5 and the ER-resident CLN6, there are similar manifestations of brain pathologies between CLN5 and CLN6 Batten disease. Human CLN5 also interacts with the ortholog of CLN7 expressed in Dictyostelium [74] and is hypothesised to be functionally related to CLN8 [75], however, further research is required to understand the interaction between CLN5, CLN7 and CLN8.

Apart from NCL proteins, a number of interacting partners of CLN5 have been reported. CLN5 co-immunoprecipitates the lysosomal sorting receptor sortilin, Rab7 and Rab5 but not Rab1a [76]. Based on the data identifying sphingolipids downstream of dihydroceramide synthase (CerS) in CLN5 patient fibroblasts [at the time incorrectly described as CLN9 variant [77]], the interaction of CLN5 with CerS was confirmed by Haddad et al. [76]. A co-immunoprecipitation (co-IP) against CerS1 in normal and CLN5 depleted fibroblasts found gamma-actin to be missing from CLN5 depleted fibroblasts [75]. Interestingly, the authors continued with a second co-IP against the missing protein gamma actin from normal and CLN5 depleted fibroblasts and identified 8 proteins missing: vimentin, the histone proteins H2AFZ/H3F3A/H1H4H and H2A type 2-C [75, 76]. The absence of gamma actin from the CLN5 depleted fibroblasts might explain the associated growth defects in CLN5 depleted fibroblasts observed by the authors, which can further explain the absence of these abundant histone proteins from CLN5 depleted fibroblasts [75]. More recent studies with proteomics analysis on CLN5-deficient cells have not supported these results [78]. Furthermore, a 2013 proteomic study [79] identified abundant nuclear and cytoplasmic proteins to be present as contamination in most proteomic studies, casting doubt on the results from Haddad et al. [75] (showing histones and vimentins altered in CLN5 deficient cells).

The studies described to this point were low-throughput studies, usually working with a candidate approach. CLN5 has been also studied using high-throughput approaches and at least 43 interactors were identified by affinity capture – mass spectrometry methods over 5 studies referenced on NCBI and the BioGRID (https://www.ncbi.nlm.nih.gov/gene/1203; https://thebiogrid.org/107614 [80–85] (Table 2). F-box protein 6 (FBXO6) was found in three of the studies [81–83]; odorant binding protein 2A (OBP2A), calreticulin 3 (CALR3) and lipase member H (LIPH were each found in both Huttlin et al. studies [81, 82], while all other interactors were only identified in one study each. Scifo et al. [83] performed targeted immunoprecipitation followed by mass spectrometric analysis, however, they did not report any CLN proteins interacting with CLN5. The other reports were not directed at CLN5, which could be why none of the studies reported any CLN proteins as interacting partners of CLN5. The different studies investigating CLN and non-CLN iterating partners of CLN5 used different proteomic techniques, Thus some CLN proteins could have been missed in more stringent conditions. A more comprehensive quantitative proteomics approach in human neurons is required to learn about the interacting partners of CLN5 that may address the molecular phenotypes of CLN5 Batten disease.

#### Role of CLN5 in the regulation of cellular components/processes

##### CLN5 and retromer association

Retromers are a group of proteins that form the endosomal protein sorting machinery, which returns proteins from the endosomes to the TGN or plasma membrane, avoiding lysosomal degradation [86]. Efficient targeting of lysosomal proteins from the endosome to the TGN requires trafficking of the lysosomal sorting receptors sortilin and the cation independent-MPR via the retromer complex [87, 88]. CLN5 plays a role in regulating this process by controlling the localisation and activation of the retromer-interacting GTPase Rab7 to the endosomal membrane [76]. CLN5 interacts with the lysosomal sorting receptor sortilin but does not fit the requirements for a cargo itself as CLN5 can interact with sortilin itself at acidic pH [76]. Loss of CLN5 leads to a phenotype similar to retromer-depleted cells or cells deficient in Rab7 palmitoylation, which is required for retrograde trafficking of lysosomal sorting receptors [89]. The membrane-bound fraction of the retromer component Vps26 was reduced in CLN5 depleted cells, yet increased in the cytosolic fraction, supporting a role for CLN5 in retromer recruitment to endosomal membranes. Mamo et al. showed that CLN5 knockdown in HeLa cells impairs the localisation of Rab7 and Rab5, while immunoprecipitation experiments showed interaction between CLN5, Rab7 and Rab5. Furthermore, the authors showed that CLN5 activates Rab7, but not Rab5 in HeLa cells. CLN5 indirectly interacts with Rab5 and Rab7, most likely via sortilin or CLN3 or both [76]. The binding of Rab7 to Rab-interacting lysosomal protein (RILP) was less efficient in CLN5 knockdown HeLa cells, indicating that CLN5 might be acting as a scaffold and is required to recruit and activate Rab7, which subsequently recruits retromer to endosomal membranes [76].

##### CLN5 and macrophages / lipid trafficking

Like several other neurodegenerative diseases showing disturbed lipid metabolism, CLN5 deficiency also results in an altered serum lipid profile. Serum samples collected from one-month-old *Cln5*^*−/−*^ mice had increased total cholesterol and phospholipid transfer protein activity. High density lipoprotein particles were especially elevated in *Cln5*^*−/−*^ serum, with only a slight increase in very low density lipoprotein. Macrophages are an important cell type for lipid processing in tissues. When peritoneal macrophages were isolated from *Cln5*^*−/−*^ mice and loaded with labelled cholesterol-low density lipoprotein, the uptake was not affected, but higher efflux was recorded. With sphingolipid-metabolism closely related to sterol metabolism, sphingolipid trafficking was assessed. In *Cln5*^*−/*−^ macrophages as well as fibroblasts, the sphingolipid transport from endo-lysosomes to Golgi appeared delayed [37], suggesting a direct or indirect role of CLN5 in lipid transport.

##### CLN5 and biometals

Metal dyshomeostasis is a common phenomenon in neurodegenerative diseases like Alzheimer’s and Parkinson’s disease, which could lead to oxidative stress and neuronal demise [90–92]. A study that investigated the levels of biometals in the CNS of mouse models of CLN1, 3 and 5 diseases, found that in *Cln5*^*−/−*^ mice, the levels of zinc, copper, manganese, cobalt and iron were significantly elevated (calcium, magnesium, potassium and sodium were not tested) [93]. The increase for zinc was apparent by five months and was statistically significant from seven months. Zinc increased by 34% in the hippocampus and 130% in the olfactory bulb. Copper levels were elevated in the hippocampus at five months and CNS-wide at seven months. Manganese levels continued to progressively rise over the course and were increased in the cortex, olfactory bulb and cerebellum at seven months. Iron levels were elevated in the olfactory bulb, the cerebellum and the spinal cord at seven months. Cobalt concentration in the cerebellum, cortex and olfactory bulb were the earliest metal changes detected at five months and progressively increased in every tissue except the hippocampus by seven months [93]. Hence, further research on metal transporters might reveal valuable information on metal homeostasis in CLN5 Batten disease. Whether metal transporters are affected in CLN5 deficient cells, whether metal dysregulation also occurs in other affected tissues, and whether metal dysregulation contributes to the disease manifestation and progress are all uncharted territories that need to be explored in future studies.

##### CLN5 and organelle crosstalk

Organelle crosstalk is disrupted in neurodegenerative diseases [94, 95]. Similarly, CLN5 loss causes mitochondrial dysfunction, which could be a secondary effect of organelle cross-talk. Doccini et al., performed a proteomic study using neurons-like SHSY-5Y cells, *Cln5*^*−/−*^ mice and NCL patient skin fibroblast to show that loss of CLN5 disrupts oxygen consumption, ATP production and respiratory chain enzyme activity [78]. Furthermore, CLN5 loss caused generation of reactive oxygen species and increased mitophagy that contribute to impaired synapse formation, axonal growth and neuritogenesis [78]. However, how a lysosomal protein causes mitochondrial changes are yet to be explored. Cross-talk between lysosomes and ER is well established, particularly in neurodegenerative disease [96]. Not much is known about the role of CLN5 in lysosome-ER crosstalk. CLN6, an ER-resident NCL protein, regulates the correct transport of CLN5 to the lysosome, and loss of CLN6 disrupts transport of CLN5 [59]. Mutations in four NCL genes (*CLN1, CLN3, CLN6* and *CLN8*) can mount ER stress eventually leading to apoptosis [96]. However, nothing is known in ER stress with the loss of CLN5, although CLN5 mutants have been shown to be retained in the ER.

## CLN5 human disease–mutations and pathology

To date, 58 disease-causing mutations and polymorphisms in the *CLN5* gene have been reported, which include 22 missense mutations, 10 nonsense mutations, 2 frameshifts, 9 deletions, 3 insertions and 12 sequence variants (Table 3, Fig. 1b). The first cases of CLN5 vLINCL (also known as fvLINCL) were identified by Santavuori in 1982 [3, 16] in Finland, and showed an onset between 4 and 7 years of age. The symptoms were mental retardation, blindness, ataxia, muscular hypotonia, myoclonus and epilepsy [3, 16]. A following report by the same group in second patient cohort identified additional symptoms including clumsiness, motor and cognitive decline. Reported subsequently, additional symptoms include hyperactivity, aggression, intolerance, anxiety, obsessive activities, hallucination, sleep alterations, autistic features, attention deficits and speech regression [31]. All of these symptoms manifest before 11 years of age [4, 5]. The initial Finnish patients were found to carry the Y392* (New RefSeq sequence–Y343*, Finnish major mutation, Table 3) in the *CLN5* gene, as confirmed by Savukoski et al. [16] The average life expectancy of CLN5 patients is between 10–30 years, with some patients reported to survive until 39 years of age [4].

**Table 3:** Mutations associated with CLN5 Batten disease

Reported mutations/polymorphisms			Location	Type of mutation	NCL age of onset/additional symptoms	Country of origin (number of families)	References
cDNA changes	Amino acid changes based on 407 aa sequence	Amino acid changes based on 358 aa sequence					
c.4C > T	p.R2C	–	–	Sequence variant		Argentina (6), Turkey (13), UK (1), Canada (1), Czech Republic (n.a.)	[7]
c.61C > T	p.P21S	–	–	Missense	Congenital/infantile/adult onset spinocerebellar ataxia	Turkey (1), Brazil (1), northern European (1)	[8, 9]
c.72A > G	p. =	–	–	Sequence variant		USA (1), Argentina (1)	[7, 8]
c.223 T > C	p.W75R	**p.W26R**	Exon 1	Missense	Late-infantile	Turkey (3)	[8]
c.225G > A	p.W75*	p.W26*	Exon 1	Nonsense	Juvenile	Sweden (1), Finland (1), Canada (1)	[7, 8, 16, 97]
c.234C > G	p. =	p. =	Exon 1	Sequence variant		USA (1)	[7, 8]
c.291dupC	p.S98Lfs*13	p.S49Lfs*13	Exon 1	1-bp insertion	Early onset (4 mo)	Argentina (1)	[9]
c.320 + 8C > T	p. =	p. =	Intron 1	Sequence variant	Infantile/juvenile	USA (1), Turkey (4), Cook Islands (1), Canada (1)	[7, 8]
c 321-1G > A	p. =	p. =	Intron 1	Sequence variant	Early onset (1y 5 mo)	China	[99]
c.334C > T	p.R112C	**p.R63C**	Exon 2	Missense	Juvenile	China (2)	[100]
c.335G > A	p.R112H	**p.R63H**	Exon 2	Missense	Juvenile/late infantile	Colombia (1), UK (1), China (2), India (1)	[8, 31, 100, 101] Faruq 2020 (submitted)
c.335G > C	p.R112P	**p.R63P**	Exon 2	Missense	Late-infantile	Portugal (1)	[54]
c.377G > A	p.C126Y	**p.C77Y**	Exon 2	Missense	Adult, late onset (age 17)	USA (1)	[7, 8]
c.433C > T	p.R145*	p.R96*	Exon 2	Nonsense		UK (1)	[8]
c.434G > C	p.R145P	**p.R96P**	Exon 2	Missense	Late-infantile	China (1)	[102]
c.477 T > C	p.C159R	**p.C110R**	Exon 3	Sequence variant	Late-infantile	Pakistan (2)	[103]
c.486 + 139_712 + 2132del	p.K163Qfs*11	p.K114Qfs*11	Exon 3	Frameshift	Congenital/infantile	northern European	[9]
c.486 + 5G > C	p.?	p.?	Intron 1	Sequence variant		Canada (1)	[8]
c.524 T > G	p.L175*	p.L126*	Exon 3	Nonsense		Turkey (1)	[8]
c.527_528insA	p.G177Wfs*10	p.G128Wfs*10	Exon 3	1-bp insertion	Late-infantile	Pakistan (1), USA (1)	[7, 8, 31]
c.528 T > G	p. =	p. =	Exon 3	Sequence variant		Sweden (1), Argentina	[7, 8]
c.545 T > G	p.M182R	**p.M133R**	Exon 3	Missense	Late-infantile	Canada	[104]
c.565C > T	p.Q189*	p.Q140*	Exon 3	Nonsense	Late-infantile	Portugal (1)	[54]
c.575A > G	p.N192S	**p.N143S**	Exon 3	Missense	Juvenile	USA (1)	[7, 8]
c.593 T > C	p.L198P	**p.L149P**	Exon 3	Missense		Turkey (1)	[8]
c.595C > T	p.R199*	p.R150*	Exon 3	Missense	Late infantile	Italy, Finland, UK, Bahrain, Erithrea (family not reported); China (2)	[31, 100]
**c.613C > T**	p.P205S	**p.P156S**	Exon 3	Missense	Late-infantile	Canada (2), Arab (Qatar and Yemen) (1)	[8, 105]
c.619 T > C	p.W207R	**p.W158R**	Exon 3	Missense	Late-infantile	UK (1), Italy, Finland, UK, Bahrain, Erithrea (family not reported)	[8, 31]
c.620G > C	p.W207S	**p.W158S**	Exon 3	Missense	Infantile/juvenile	China / USA (1), Finland (1)	[7, 8, 31]
c.623G > A	p.C208TY	**p.C159Y**	Exon 3	Missense	Late-infantile without visual decline	China (2)	[106]
c.669dupC	p.W224Lfs*30	p.W175Lfs*30	Exon 3	1-bp insertion	Juvenile	Sweden (1), Finland (1), Canada (1)	[7, 8, 97]
c.671G > A	p.W224*	p.W175*	Exon 3	Nonsense	Late-infantile	USA (2), UK (1)	[7, 8, 31]
c.694C > T	p.Q232*	p.Q183*	Exon 3	Nonsense	Juvenile	Serbia (1)	[75]
c.718_719delAT	p.M240Vfs*13	p.M191Vfs*13	Exon 4	2-bp deletion	Late-infantile without visual decline	China (2)	[106, 107]
c.726C > A	p.N242K	**p.N193K**	Exon 4	Missense		UK (1), Turkey (2), Brazil (1)	[8]
c.741G > A	p.W247*	p.W198*	Exon 4	Nonsense	Late-infantile, no cerebral atrophy	Iran (1)	[108]
c.741_747delinsTT	p.W247Cfs*5	p.W198Cfs*5	Exon 4	Frameshift	Late-infantile	Middle East (1)	Mutation was only mentioned in [102] and NCL database
c.755-756insC	p.E253*	p.E204*	Exon 4	Insertion	Late-infantile	Sweden (1)	[32]
c.772 T > G	p.Y258D	**p.Y209D**	Exon 4	Missense	Juvenile, seizures later	Italy (1), Italy, Finland (family not reported)	[8, 31, 109]
c.788 T > A	p.V263E	**p.V214E**	Exon 4	Missense	Late-infantile	Italy, Finland, UK, Bahrain, Erithrea (family not reported)	[31]
c.835G > A	p.D279N	**p.D230N**	Exon 4	Missense	Late-infantile	The Netherlands (1), Portugal (1)	[16, 54, 97]
c.907_1094del188	p.T303Cfs*10	p.T254Cfs*10	Exon 4	deletion	Juvenile, adult	USA (1)	[7, 8]
c.919delA	p.R307Efs*29	p.R258Efs*29	Exon 4	1-bp deletion	Juvenile	Egypt (1), USA (1)	[7, 8]
c.925_926del	p.L309Afs*4	p.L260Afs*4	Exon 4	Sequence variant	Late-infantile	Pakistan (2)	[103]
c.935G > A	p.S312N	**p.S263N**	Exon 4	Missense	Adult onset	Italy (1)	[110]
c.955_970del16	p.G319Ffs*12	p.G270Ffs*12	Exon 4	16-bp deletion		UK (1)	[8]
c.959A > G	p.N320S	**p.N271S**	Exon 4	Sequence variant	Associated with Alzheimer's disease, no NCL phenotype reported	Carribean Hispanic (1)	[11]
c.1026C > A	p.Y342*	p.Y293*	Exon 4	Nonsense	Late-infantile	Roma from the former Czechoslovakia (1)	[111]
c.1054G > T	p.E352*	p.E303*	Exon 4	Nonsense	Late-infantile	Newfoundland / UK (1)	[112]
c.1071_1072delCT	p.L358Afs*4	p.L309Afs*4	Exon 4	2-bp deletion	Late-infantile, late visual symptom	China / USA (1)	[7, 8, 99]
c.1072_1073delTT	p.L358Afs*4	p.L309Afs*4	Exon 4	2-bp deletion	Early juvenile	Pakistan (1), UK (1)	[8, 59]
c.1082 T > C	p.F361S	**p.F312S**	Exon 4	Missense	Late-infantile without visual decline	China (2)	[106]
c.1083delT	p.F361Lfs*4	p.F312Lfs*4	Exon 4	1-bp deletion	Juvenile	USA (1)	[7, 8]
c.1103_1106delAACA	p.K368Sfs*15	p.K319Sfs*15	Exon 4	4-bp deletion	Juvenile	USA (1), Spain (1)	[7, 8, 99, 113]
c.1103A > G	p.K368R	**p.K319R**	Exon 4	Sequence variant	Late-infantile	USA (1), Finland (family not reported), Argentina (family not reported.), Turkey (4), Canada (5), India (1), Hispanic (1)	[7, 16]
c.1121A > G	p.Y374C	**p.Y325C**	Exon 4	Missense	Adult (age 17)	USA (2)	[7, 8]
c.1137G > T	p.W379C	**p.W330C**	Exon 4	Missense	Late-infantile	Afghanistan (1)	[59]
c.1137G > A	p.W379*	p.W330*	Exon 4	Nonsense	Infantile, early motor symptom, late seizures	Italy, Finland, UK, Bahrain, Erithrea (family not reported)	[31]
c.1175_1176delAT	p.Y392*	p.Y343*	Exon 4	2-bp deletion	Late-infantile	Finland (19), Italy, Finland, UK, Bahrain, Erithrea (family not reported)	[16, 31, 97]
c.*33A > G (formerly 1224 + 33A > G)	p. =	–	3′ UTR	Sequence variant		USA (1)	[7]

Bolded texts refer to the changes in the *CLN5* protein due to the mutations in the current updated *CLN5* gene

In the 1990s, Savukoski et al*.* first reported the Fin major, Fin minor and Fin European (in a Dutch patient) mutations in *CLN5* (Table 3). Several other mutations in CLN5 were reported from different parts of the world over the past 30 years. Later studies found additional *CLN5* mutations in other European countries, the United States, Canada, south America, the Middle-East, China, India and Pakistan [7–9, 11, 16, 31, 32, 54, 59, 75, 97–113] (Table 3, Fig. 1b). The identification of more CLN5 patients also highlighted differential disease progression. For example, juvenile forms of the disease in Colombian patients (R112H, (New RefSeq sequence–R63H) (Table 3) show onset of symptoms after the age of 9 years [101], whereas only one report from Argentina accounts for onset of symptoms as early as four months of age, suggesting the existence of an infantile form of CLN5 Batten disease [9]. Do the differential mutations dictate how CLN5 is dysregulated, not just in the brain but in peripheral tissues, and lead to associated pathologies? Do the mutations dictate the age of onset? These unanswered questions are difficult to understand without knowing the function of CLN5.

Neuronal demise in the cerebrum and the cerebellum are key features of CLN5 Batten disease pathology. Magnetic resonance imaging (MRI) in affected children between 6–11 years of age revealed that cerebellar atrophy is one of the leading signs at the time of diagnosis, while a later study showed cerebral atrophy in affected children between 6.6–17.4 years of age [97, 114]. Macroscopic analysis of fvLINCL brains showed thinner cerebral cortex, reduced white matter and enlarged ventricles [45]. In advanced stages, loss of cortical neurons is observed in layers III and V, accompanied by severe cerebral cortical astrocytosis [44, 45]. Moderate-to-severe loss of myelin and gliosis are also observed in cerebral white matter. Cerebellar purkinje cells and neurons in the granular layer of the cerebellar cortex are destroyed [45]. This is accompanied by astrocytosis and an increase in glial cells in the cerebellum. Neurodegeneration is also shown in the thalamus, in addition to the loss of large striatal neurons, nigral and spinal motor neurons [45]. Retinal dystrophy and pan-retinal degeneration are considered to be earlier pathologies in CLN5 Batten disease. Bull’s eye maculopathy, diffuse pigmentary degeneration, arteriolar attenuation and optic atrophy develop with the progression of disease [115]. These phenotypes have also been confirmed in animal models of CLN5 Batten disease, discussed in section “Tissue expression of CLN5” [26].

A common feature in NCL-affected neuronal cells is accumulation of autofluorescent storage material (ASM) in the lysosomes, a key hallmark of all Batten disease variants. Ultrastructural investigation of the ASMs in fvLINCL showed fingerprint, curvilinear and rectilinear bodies [3, 7, 45, 101] as compared to granular osmophilic deposit (GROD) lipopigment and fingerprint morphology observed in infantile and adult forms of the disease [116, 117]*.* The main constituent of this lysosomal storage material was found to be subunit C of mitochondrial ATP-synthase, while lipids are found as minor components with profiles attributable to lysosomal/endosomal origins [45]. In addition to the ASM found in the brain cells, peripheral cells also show similar deposits. Cardiomyocytes, skin eccrine glands, peripheral neurons and parietal cells in gastric mucosa show extensive deposits, whereas hepatocytes, smooth muscle cells, kidney tubules, adrenal cortex, adipocytes, thyrocytes and pancreatic cells show less storage material accumulation [48]. It is still unclear whether the accumulation of storage materials leads to neurodegeneration.

Lysosomes, the cellular waste recycling machinery, require an acidic environment for the effective degradation of cellular substrates. In fibroblasts derived from CLN5 Batten disease patients, the lysosomal pH is significantly higher than control fibroblasts, without changing the cytoplasmic pH [118]. This phenotype has also been identified in the CLN5 sheep and mice models [10, 26]. Our group has previously shown autophagic impairment in CLN5 ovine cultures [10], however, it is still not clear if lysosomal pH change leads to autophagy defects or autophagy impairment leads to changes in lysosomal acidity. An elevated pH will render the lysosomal enzymes dysfunctional, resulting in improper degradation and clearance of waste material delivered to the lysosome. It is generally believed that the loss of the major lysosomal function leads to the accumulation of storage materials. In neurons, in addition to degradation and recycling function of lysosomes, they regulate neuronal health and development, synaptic activity and RNA molecule transport [119–124]. Hence, elevated lysosomal pH might impair one or all of these lysosomal regulatory roles as well. Our previous study has indicated defects in bulk synaptic endocytosis in a CLN5 ovine neural culture model [10]. One report suggested that impaired mitophagy and multiple defects in the activity of respiratory chain enzymes in the cerebral cortex is also associated with CLN5 Batten disease, suggesting mitochondrial defects in addition to the lysosomal impairment [78]. Whether mitochondrial dysfunction is a primary or a secondary effect due to loss of CLN5 is still unclear.

Given the post-mitotic nature of neurons, waste clearance is even more critical in these cells than in dividing cells. Nonetheless, CLN5 deficiency-related phenotypes are not just restricted to neurons, but have the potential to have far-reaching effects in other cells/organs, including the heart. Hence, targeting multiple cell types is key for developing a treatment or cure for CLN5 Batten disease. With CLN5 expression being fairly high in several peripheral tissues, and the effect of loss of CLN5 still unknown in most of these tissues, a cautious approach needs to be taken while developing therapies to treat CLN5 Batten disease.

## Model organisms used to study CLN5

**Fig. 3:**
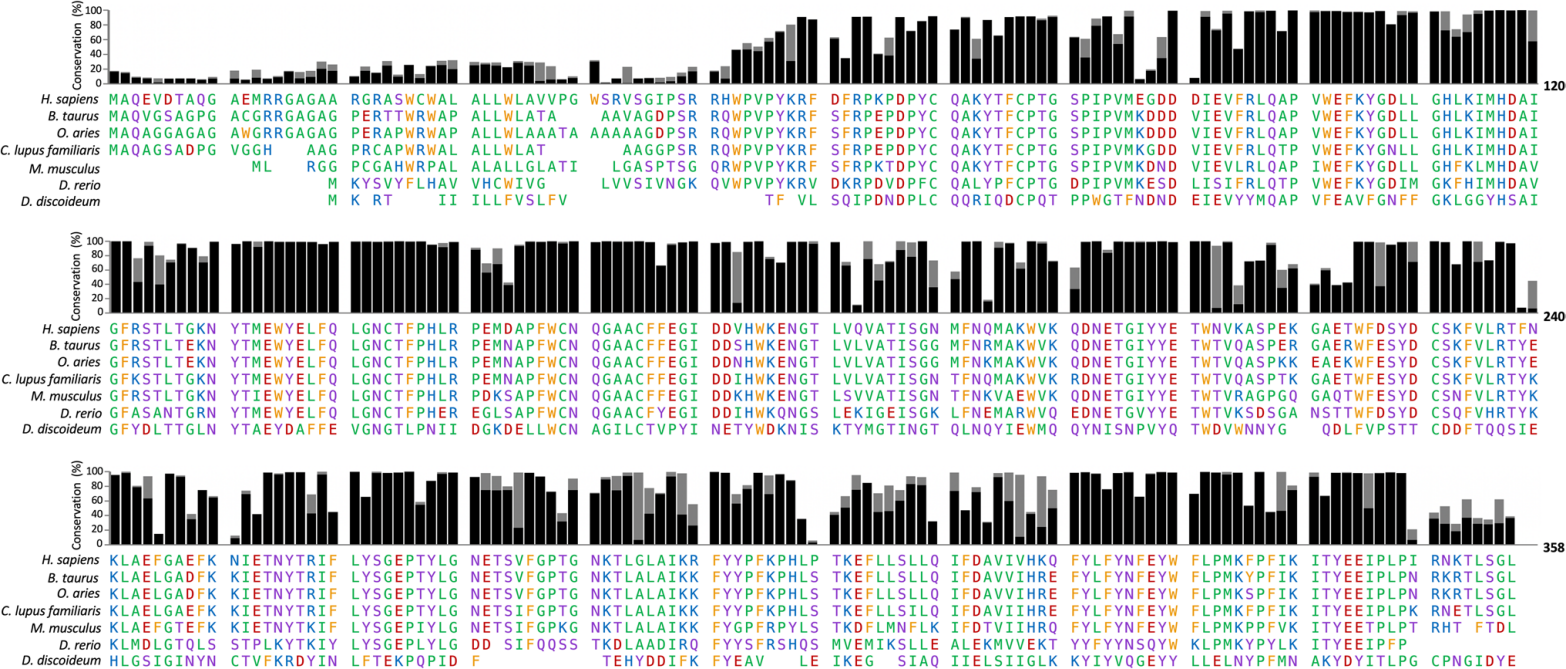
Mature CLN5 is highly conserved throughout species. Black bars represent the percent conservation of each human CLN5 residue across the 397 CLN5 protein sequences available on UniProt. Stacked grey bars represent the percent conservation of amino acids with similar chemical properties. Underneath are the aligned amino acid sequences for CLN5 from the model organisms used to study CLN5. Amino acids are coloured according to their chemical properties: non-polar residues (G, A, V, C, P, L, I, M) are in green; polar, uncharged residues (S, T, Y, N, Q) are in purple; basic, positively charged residues (K, R, H) are in blue; acidic, negatively charged residues (D, E) are in red; and aromatic residues (W, F) are in yellow. Created using Microsoft Excel and Powerpoint

As is the case for many forms of Batten disease, the lack of knowledge surrounding CLN5 function severely hampers the development of prospective therapies. The development and use of robust model systems, from single cells to large animals, is critical to advancing our understanding of CLN5 disease pathology, identifying novel therapeutic targets and developing therapies. Several model organisms came into the limelight due to the naturally occurring mutations in CLN5. In conjunction with genetically modified organisms, CLN5 model systems range from multicellular social amoeba and in vitro cell culture models, to small (mouse) and large (sheep, cattle and canines) vertebrates. With similar pathologies to those observed in humans and a conserved CLN5 sequence (Fig. 3), these models are key to understanding the underlying biology of CLN5 Batten disease. We have provided details on the systems used for modelling CLN5 disease and highlighted the recent advances that have been achieved through the use of these models (Table 4).

**Table 4:** Advantages and disadvantages of model systems used in CLN5 research

Model System	Model Species	Gene Mutation	Use in pathology research and/or therapy development	Advantages	Disadvantages
Large Animal Models	**Cattle** - Australian Devon	CLN5:c.662dupG	- Original Description only	- Pathological and behavioural similarities to human disease	- Lack of further characterisation beyond original description
	**Sheep** - Borderdale (NZ)	CLN5:c.571 + 1G > A	- Blindness, cognitive and behavioural changes - Cortical atrophy - ASM accumulation - Primary neural cultures	- Closer to human in size and pathology manifestation - Gene therapy testing	- Gene therapy testing did not prevent blindness - Expensive and time-consuming
	**Canine** - Border Collie - Golden Retriever - Australian Cattle - Australian Cattle-German Shepherd - Unknown breed	CLN5:c619 C > T CLN5:c934_935delAG CLN5:c619 C > T CLN5:c619 C > T CLN5:c619 C > T	- Original descriptions - Biomarker identification	- Many of the behavioural and pathological phenotypes resemble human pathology - Has been great success with dog models for therapy development in CLN2	- Some behavioural phenotypes are more pronounced than seen in human patients - Increased time and costs to establish as a colony
Smaller Animal Models	**Murine** - Cln5 knockout mouse	*Cln5* knockout	- Original description - Retinal pathology - Glial pathology - Mitochondrial pathology - Neurogenesis - Primary neural cultures - Lipid metabolism - Biomarkers	- Mouse models are cost effective, easy to use, have a relatively short lifespan and are useful for therapeutic testing in a whole organism - Cln5 model has provided insights into disease pathogenesis and potential CLN5 functions	- Lack of *Cln5* model characterisation with respect to behavioural phenotypes and disease course - Therapies tested in mice often fail to translate well to human application -Often fail to completely recapitulate human pathology
	**Zebrafish**	N/A	- One CLN5 homolog identified	- Useful model for studying developmental genetics and screening for drugs	- To date, no experiments looking at CLN5 have been published
Small Eukaryotic Models	***Dictyostelium discoideum***	Cln5 knockout	- Original Descriptions - CLN5 interactions - Secretion - Mitochondrial (dys)function - Autophagy	- Recapitulates ASM phenotype in human CLN5 disease - Provided insight into CLN5 pathogenesis and function - Can study CLN5 in the context of a whole organism - Cost effective and easy to maintain	- Organism lacks a nervous system
I*n vitro* systems	**Human** - Cell lines - Patient-derived fibroblasts - Induced pluripotent stem cells (iPSCs) Non-Human - Primary ovine neural cultures - Primary murine neural cultures	CLN5 knockout iPSC CLN5^y392X^ CLN5:c.571 + 1G > A in sheep Cln5 knockout in mouse	- Trafficking - Post-translational modifications Interactions - Lysosomal acidity - Autophagy - Endocytosis - ASM accumulation	- Time and cost efficient - Highly amenable to gene editing techniques - Good for studying cell type specific CLN5 pathophysiology - Recapitulate the human phenotype - Fibroblasts and iPSC-derived neurons reflect patient age and environmental etiopathology - Ideal for drug screening and personalised medicines, like screening of gene therapy candidates	- Does not recapitulate disease at the tissue or organism level - Immortalised cell lines are not pure populations and have cancer characteristics - Cells are grown as a monolayer which does not represent the brain environment - Cell cultures often do not show ASM accumulation

### Small animal models

Among small animal models, mouse models are well established and commonly used in the study of human neurological conditions. Owing to their ease of use, short lifespan and breeding cycles, the variety of established mouse-based research tools and their proven physiological phenotypes/similarity with the human genome they provide a powerful tool to test preclinical therapies and investigate disease pathogenesis.

#### Mice

The mouse *Cln5* gene encodes a 341 aa protein, shares 74% identity with human CLN5 (Fig. 3), is expressed throughout the mouse brain (discussed in section “Tissue expression of CLN5”) and has five N-glycosylation sites (N113, N161, N186, N238 and N254) [125]. The *Cln5*^*−/−*^ mouse model was developed through the insertion of a neomycin cassette into exon 3 of the *Cln5* gene resulting in a truncated protein [46]. The first pathological symptom in mice, congruent to that in humans, is progressive visual loss beginning at 13 weeks of age, indicating neurodegeneration in the visual system [46]. Characteristic ASM accumulation is present in the retina and the brain, with cortical neurons displaying the fingerprint lamellar profile. From four months of age, neuronal loss becomes first apparent in the cortex, as opposed to the thalamocortical regions in mouse models for other NCLs [46, 47]. GABAergic interneuron loss is seen in multiple brain regions by six months and brain atrophy seen by twelve months [46, 47]. In *Cln5*^*−/−*^ mice, increase in proliferation of neural progenitor cells (NPC) is accompanied by impairment in NPC migration, however, the mature neurons are generated with normal morphology [36, 38]. Adult mice also displayed cortical hyperexcitability and had decreased numbers of hippocampal parvalbumin-positive interneurons [126]. In both *Cln5*^*−/−*^ embryos and primary cultures there are deficits in neuronal differentiation and the development of interneurons, highlighting the potential for a much earlier development of CLN5 disease than previously thought.

In *Cln5*^*−/−*^ mice astrocytosis and microgliosis starts from one month of age and even before the observed neurodegeneration [37]. The expression of CD68, a marker for activated microglia, is already higher in the somatosensory cortex and ventral posterior nucleus of the thalamus by three months of age in *Cln5*^*−/−*^ mice. This increased CD68 expression is also widespread to other CNS structures, such as other thalamic relay nuclei, cortical regions, subiculum, selected hippocampal subfields, globus pallidus and substantia nigra. Staining with Iba-1, a marker for ramified/resting as well as activated microglia, showed wildtype cells with long, branched processes and small cell bodies, whereas *Cln5*^*−/−*^ cells showed darker stained microglia with a larger cell soma and fewer branched processes, indicating the ongoing microglial activation at an early age (one month). When cultured, *Cln5*^*−/−*^ microglia were still capable of mounting an immune response, as they secreted similar amounts of cytokines as compared to wildtype cells [37]. As discussed in section “CLN5 and macrophages / lipid trafficking”, Cln5 deficiency showed altered lipid metabolism, in addition to sphingolipid transport and defective myelination prior to neurodegeneration [37].

While studying the retinal pathology in the *Cln5* mouse model, electroretinography revealed signs of pathological events in the retina from one month of age. Progressive decline in retinal function is observed alongside photoreceptor apoptosis, ASM accumulation, glial infiltration and increased autophagy. Rod photoreceptor-mediated functional decline occurs earlier followed by late stage cone photoreceptor-mediated functional decline. The observed vision loss in mice is primarily due to photoreceptor degeneration and is postulated to be due to failure of the lysosome to degrade phagosomes and autophagosomes. No optic nerve injury was reported [26].

While the only known *Cln5* mouse model has advanced our understanding of disease pathogenesis, there are still significant gaps in behavioural phenotypes, disease course and endpoint manifestation as compared to the human disease. Humans and mice significantly differ in size, longevity, brain structure and mental function which likely underpins why many of the therapies tested in mice do not translate well to human application.

### Large animal models

Large animal models are more comparable to humans than mouse models in terms of pathophysiology, size, lifespan, genetics, CNS structure and anatomy. This makes them essential in bridging the gap between ‘simpler’ models and patients, both in terms of understanding disease pathogenesis and testing therapeutics. Naturally occurring CLN5 mutations have been identified in sheep, dogs and cattle (Table 4). However, many of these are just case reports with no further research, which provides potential opportunities for future work.

#### Sheep

The ovine CLN5 has 91% homology with the human CLN5 (Fig. 3). Naturally occurring CLN5 disease in the New Zealand Borderdale sheep was first reported in 2002 [127]. These sheep were identified as displaying NCL-like pathology which was subsequently confirmed to be due to a nucleotide substitution in CLN5 resulting in a truncated protein (CLN5:c.571 + 1G > A [128]). The CLN5 Borderdale sheep are excellent models of the human disease, showing many similarities in clinical progression and pathology. Like in their human counterparts, the first clinical symptom is blindness, presenting from ten to eleven months, followed by progressive cognitive decline and behavioural changes, culminating in death at around 2 years old [127, 128]. These symptoms are accompanied by classical pathologies, including ASM accumulation and advanced cortical atrophy as well as synaptic alterations throughout the motor cortex [129]. As discussed previously, advanced cortical atrophy is a major pathology observed in humans, but is absent from the mouse model, emphasising the importance of the sheep model. Further characterisation combining electromyography, electrooculography and electroencephalography measurements identified sleep abnormalities, as has been reported in both human and dog CLN5 disease [130–132].

The New Zealand Borderdale sheep flock has been utilised for gene therapy testing since 2008. Initial work focusing on vector optimisation showed relatively low levels of transduction with lentiviral mediated gene delivery limited to near the intracortical injection site [133]. However, this was sufficient to attenuate CLN5 disease progression [134]. The use of AAV9 vectors produced widespread expression from a single injection site and, as was seen for the lentiviral therapy, prevented the development of CLN5 disease pathology and behavioural deficits [134]. While promising, this work was carried out pre-symptomatically, which doesn’t reflect what would likely be currently possible for human patients. As the majority of human NCL patients are diagnosed following the onset of disease symptoms, a follow up study was carried out in sheep with established clinical disease (seven months age). When given after disease onset, AAV9-CLN5 treatment was able to stabilise the disease [134]. Furthermore, extensive characterisation of this flock has led to a battery of biomarkers including, but not limited to, behavioural tasks, clinical measurements, computed tomography scan, electroencephalogram and electroretinogram [134, 135]. These biomarkers will be essential for future work in testing and optimising therapeutic strategies. This work highlights the usefulness of this model in establishing the natural history of the disease and identifying biomarkers in order to analyse therapeutic doses, vector distributions and track therapeutic efficacy. To this end, although the results from these trials are very promising, the treatments were not able to prevent blindness in the post-symptomatically treated sheep [134]. It is likely that the vectors did not target or persist long enough in, the retina to prevent the observed retinopathy. This highlights the need for further therapeutic optimisation and the likely need for combinatorial treatments with simultaneous retinal targeting.

####  Dogs

The canine CLN5 has 92% homology with the human CLN5 (Fig. 3) and naturally occurring CLN5 mutations have been identified in several breeds of dogs. The most common mutation, CLN5:c619 C > T[136], was first identified in Border Collies. This same mutation has since been identified in Australian cattle dogs [50], a mixed breed Australian Cattle-German Shepherd [132] and most recently a mixed breed dog of unknown parentage [132]. A different CLN5 disease causing mutation, CLN5:c934_935delAG, has also been identified in Golden Retrievers [137].

Affected dogs show symptoms from as early as six months, with obvious and progressive neurological decline by twelve months and progressive visual loss by eighteen months, ultimately culminating in death by 3 years [132, 138]. Affected dogs display many of the symptoms and pathologies seen in human patients including, but not limited to, progressive cognitive and visual decline, seizures, brain atrophy and accumulation of ASM. Some of the affected dogs have shown aggressive behaviour, which are not as pronounced in human patients [132, 138].

#### Cattle

The cattle *CLN5* gene shows 89% homology (Fig. 3) to the human gene and encodes a 358 aa protein [139]. A naturally occurring *CLN5* mutation has been identified in Australian Devon cattle. This mutation (c.662dupG) causes premature termination and is predicted to result in a truncated CLN5 protein. Cattle with this mutation show pathological similarities to human CLN5 disease with ASM accumulation, cortical atrophy, progressive visual impairment, behavioural abnormalities and premature early death [139]. Since this initial characterisation, no further studies using cattle have been published.

### Cellular models of CLN5 Batten disease

Cellular models of CLN5 Batten disease have been invaluable tools to understand the underlying molecular pathology and range from ancient social amoeba *Dictyostelium discoideum*, to immortalised cell lines, patient derived fibroblasts and iPSCs and primary neural cultures from mice and sheep (Table 4). These models can be genetically manipulated with commonly used molecular biology techniques including site directed mutagenesis and CRISPR/Cas9 to generate specific mutations to understand CLN5 protein biology. Non-human derived models have human orthologs of CLN5 with similar functions. However, simple model organisms for CLN5 are restricted to *Dictyostelium* with no orthologs in yeast, Drosophila or *C. elegans*. When compared with animal models, cellular models are cost effective and easy to maintain and manipulate. Furthermore, cellular models can be used to test cell-type specific effects of potential treatments. It is due to the aforementioned advantages that cellular models provide a useful tool for preliminary screening of potential therapeutics. However, cellular models must be used in conjunction with animal models to validate potential treatments, as cellular models cannot test pharmacokinetics and pharmacodynamics of potential treatments. Below we describe cellular models used in the study of CLN5 Batten disease.

#### Multicellular social amoeba

*Dictyostelium* has a 24-hour life cycle, with separate cell division and development processes [140, 141] making these features valuable to study development and growth processes individually. Furthermore, non-lethal mutants that affect development pathways, while leaving cell growth unchanged, can be isolated and studied [142]. Another advantage of using *Dictyostelium* over a cell culture model is that the social amoeba provides the opportunity to study disease in a whole organism that has a 24-hour asexual single and multicellular cell life with the capability to study developmental and cellular processes, including lysosomal pathways [143].

*Dictyostelium* has a fully sequenced and annotated 34 MB haploid genome comprising six chromosomes, which encodes approximately 12,500 proteins [143–145]. The CLN5 ortholog in *Dictyostelium*, encoded by the *Cln5* gene, is a 322 aa, 37 kDa protein. Sequence homology between human CLN and *Dictyostelium* highlights a similar 301 aa region, with 30% of the aa conserved (Fig. 3). Interactome studies of Cln5 reveals interactions with numerous proteins including lysosomal enzymes (β-galactosidase and α-mannosidase), cysteine proteases and other NCL proteins; TppB/Cln2, CtsD/Cln10 and CtsF/Cln13. Cln5 secretion is influenced by Cln3 and therefore has a strong interaction with proteins linked to Cln3 function such as AprA, a quorum sensing protein, and CadA, a calcium-dependent cell adhesion protein [58].

Although *Dictyostelium* does not have a nervous system, cells that lack Cln5 show accumulation of ASM and autophagic vacuoles, pathologies seen in human CLN5 Batten disease [58]. Glycosylated Cln5 in the ER is transported to the cell cortex where it is secreted through an unconventional pathway, whereas in human CLN5 the presence of a signal peptide promotes secretion [58]. Cln5 secretion is reduced when autophagy is induced, suggesting an autophagy link with Cln5 secretion also observed in mouse models [58]. In the early stages of *Dictyostelium* development, reduced expression of Cln5 leads to reduced cell–cell adhesion, which is further intensified under autophagic conditions—another similarity with the Cln5 mouse model. Research by Huber and colleagues expressed human CLN5-GFP and *Dictyostelium* Cln5-GFP in the amoeba cells, immunoprecipitated the proteins and performed glycoside hydrolase activity assay, hence concluding that both the CLN5s are hydrolases [58]. Leubben et al. (unpublished) (http://www.rcsb.org/structure/6R99) have solved the structure of CLN5 and have predicted the human protein to be a lysosomal protease. However, it is yet to be shown whether the human CLN5, isolated from human cells, shows hydrolase or protease activity.

#### Cell culture models

A large body of work relating to CLN5 protein function and localisation has been performed in immortalised cell culture models. Human immortalised cell lines are an advantageous model in studying CLN5 disease as they are both time and cost efficient and easy to handle. These cells are also highly amenable to gene editing techniques, making them ideal candidates to study particular CLN5 pathogenic mutations. A variety of immortalised cell types have been used in the study of CLN5 Batten disease. Below we highlight studies that have linked cellular findings to human pathology.

##### Immortalised cell lines

Studies comparing tissue specific CLN5 expression with immortalised cell lines have used specific cell lines; A431 (skin epidermis), HEK293 (embryonic kidney), HeLa (cervix), HepG2 (liver), HT1080 (connective tissue) and SH-SY5Y (neuroblastoma) [35]. HEK293 and HeLa cell lines have served as useful tools to understand CLN5 trafficking, post-translational modification and protein–protein interactions, as discussed in section “CLN5 post-translational modifications, processing, trafficking and protein interactions”. Using SH-SY5Y stable lines, the interactomes of CLN3 and CLN5 revealed that both CLN variants share a common disease pathway [70, 83]. Recently, Doccini and colleagues demonstrated that SH-SY5Y CLN5 KO cells exhibit mitochondrial dysfunction and increased autophagy [78]. However, there have been concerns highlighted for the use of the SH-SY5Y cell line for the study of neurological disorders [146]. The SH-SY5Y cell line is not a pure neuronal cell line as it is immortalised from a neuroblastoma. Although these immortalised cell lines have served as invaluable tools to understand CLN5 biology, the cancerous properties of these cell lines separate the physiological characteristics from a pure neuronal population which may influence differentiation, cell viability, growth, genomic stability and metabolism.

##### Fibroblasts

Human fibroblasts represent a better model for understanding biology when compared with immortalised cell lines showing cancerous nature. Patient derived fibroblasts alongside healthy primary fibroblasts have allowed researchers to study naturally occurring CLN5 pathogenic mutations on a defined genetic background that show peripheral phenotypes [12, 35, 78]. These fibroblasts have been used to study protein trafficking, processing, stability and activity of lysosomal enzymes. Obtaining fibroblasts is relatively easy without the need for invasive procedures and they represent the patient age as well as their environmental etiopathology. Histological studies characterising CLN5 Batten disease used human fibroblasts from affected individuals to describe key pathologies of CLN5 Batten disease, including accumulation of intralysosomal inclusion bodies [31]. Fibroblasts deficient in CLN5 have dense material accumulated in vacuoles and lysosomes. Furthermore, an increase in the expression of p62 leads to inhibition of autophagosome–lysosome maturation, indicating that CLN5 is crucial for normal cell function.

The key disadvantage of patient-derived fibroblasts is that they cannot mimic cell-specific phenotypes observed in neurons (largely neurodegeneration) or cardiac cells (contractility) due to inherent differences between somatic cell types in metabolic processes. The drift in cell types in culture over passages, clonal variations and stark differences in the gene expression profiles and signalling mechanisms between fibroblasts and neurons limit their potential to accurately understand CLN disease pathologies and screen for potential therapeutics in fibroblasts. Moreover, the controls used for some of these studies were commercial fibroblasts, which were not age and sex matched for the CLN5 patient-derived fibroblasts. However, their potential in deriving iPSC-mediated neuronal differentiation or direct reprogramming into human neurons gives the fibroblast an absolute advantage over immortalised cell lines.

##### Induced Pluripotent Stem Cells (iPSCs)

iPSCs have the potential to revolutionise the way we study CLN5 disease, but they are still in the early stages of experimentation. Uusi-Rauva and colleagues reported the first CLN5 iPSC model [147]. Fibroblasts from a CLN5 patient were reprogrammed via expression of SOX2, OCT3/4, KLF4 and MYC to generate the CLN5Y^392*^ (New RefSeq sequence: Y343*) iPSC line, the predominant mutation in CLN5 Batten disease. This model can be differentiated to mature neurons, with the expected morphology which recapitulates key pathologies of CLN5 Batten disease including accumulation of ASM, changes in lysosomal structure and abnormal sphingolipid transportation. The study by Uusi-Rauva and colleagues did not report an isogenic control for the CLN5^Y392*^ (New RefSeq sequence: Y343*) iPSC cell line. Many study designs use unaffected family members for controls. However, family members are not suitable controls as they are not genetically identical. Single Nucleotide Polymorphisms (SNPs) are often dismissed despite the fact that SNPs can contribute to or dictate a disease phenotype. Future studies can be designed with either (1) wild type iPSCs and introducing the human mutation(s) into the iPSCs or (2) rectifying human mutation in iPSCs from patients, both using CRISPR technology. Both these techniques will ensure an isogenic control for the CLN5 mutation. Ideally, multiple iPSC lines with the same mutation from multiple patients can be generated using the second approach, which will provide a battery of excellent tools to analyse inter-individual variation in the disease.

A couple of caveats with iPSCs may limit their application in neuroscience, albeit depending on the research question. Tissue specific methylation, in the iPSCs, may not be properly corrected during reprogramming or may be carried through the passaging stages, affecting terminal differentiation of specific cell types [148]. A second caveat in the human iPSC model is that it is grown as a monolayer of cells, which does not represent the human brain environment. iPSCs have successfully been differentiated into brain organoids [149] that have been used as a model for CLN3 Batten disease [150]. Although these are not fully defined organs, they represent the brain better than monolayer cells, providing a more synonymous environment to diseased human organs.

##### Primary neural cultures

Limited work has been done on primary cell cultures from CLN5 ovine and mouse models. Primary cultures are an inexpensive, easy model to maintain. They are critical for understanding pathophysiology and disease progression in CLN5 disease, as well as screening for potential therapeutic compounds. Like other cellular models, primary cultures are not able to fully recapitulate the human phenotype and cannot be used to study CLN5 Batten disease at the organism level.

Primary cultures of neurons, neuroblasts, astrocytes and microglia derived from CLN5 sheep (discussed under “Sheep” in “Large animal models” section) exhibit hallmark features of Batten disease including a decrease in lysosomal acidity, autophagy and endocytosis [10]. In the study conducted by Best et al. [10], the CLN5 ovine neurons were physiologically smaller than wildtype neurons, but the authors did not comment on whether the cells accumulate ASM. The accumulation of ASM is a late onset phenotype and the sheep may not have been old enough to see altered ASM in the neurons. However, our group has previously observed that ASM was not apparent at neuronal culture plating, but appeared within a week of culturing cells from older foetuses [1]. Gene therapy replenishing wildtype CLN5 in the neural cultures reduced ASM accumulation, demonstrating that ovine cultures are a good model to study both CLN5 pathophysiology and to screen therapeutics.

Besides the ovine primary neural cultures, mouse primary neural cultures have been indispensable for our understanding of the disease pathology. Mixed mouse primary neural cultures of cortical neurons, oligodendrocytes, astrocytes and microglia have been used to show that Cln5 has higher expression in glial cells than neurons, and that astrocytes exhibit markers of cell death before neurons do [37]. In mixed mouse hippocampal neurons, Cln5 colocalised to the lysosome [34]. Cortical neurons from *Cln5*^*−/−*^ mice showed a differential distribution of proteins involved in the cytoskeleton and growth cone [47]. These primary cultures are excellent tools to study CLN5 pathophysiology, however, none of these studies have commented on whether these cultures exhibit hallmark features of CLN5 Batten disease. Hence, proper characterisation of the mouse primary cultures is essential to ensure recapitulation of human disease phenotypes, including ASM accumulation, lysosomal acidity, autophagy and synaptic endocytosis.

In considering all of these models, be it whole organisms or cellular models, it is important to remember that it is not a ‘one size fits all’ scenario. Each model comes with its own set of advantages and disadvantages (Table 4), which need to be carefully considered both when selecting a model system for experimental analysis and interpreting results. Saying that, the amount of knowledge obtained from studying these models has advanced CLN5 research to an extent where we are close to developing gene therapy to treat the disease. Hence, a combinatorial approach with cellular and animal models may represent the best way forward, with delivery mechanism, long term efficacy and safety investigation in large animal models still being irreplaceable before undertaking a clinical trial.

## Future directions

Since the discovery of mutations in vLINCL families in 1998, *CLN5* has been intensively studied both to understand its function and its potential as a therapeutic for CLN5 Batten disease. The story is still far from complete, 22 years on. In this review, we have highlighted the key findings, controversies over protein localisation and processing, human clinical and pathological symptoms, model systems and progress towards therapy. This is an exciting time to be involved in neurodegenerative disease research, and particularly rare disease research, where clinical trials are fast-tracked and the families, researchers, clinicians and biotechnology companies are joining together towards finding a cure.

CLN5 Batten disease is ultra-rare and as such models are essential in establishing treatments. The limited pool of patients that can be recruited for any one clinical trial means that we must provide the best possible evidence for efficacy and function. Knowing how CLN5 mutations cause Batten disease and having the right models and readouts to test efficacy is vital. Variation in disease progression, even between siblings, suggests a role for genetic modifiers in Batten disease. The significance and identification of such modifiers, however, remains to be established.

The CLN5 field has a strong array of model systems which are adept to test both protein function, pathologies and high-throughput screens of potential therapeutics. The recent developments in CRISPR-Cas9 and iPSCs will allow many of these exploratory studies to be completed in human-derived neural cells. Patient-derived iPSCs must be complemented with isogenic controls, corrected for the mutation to control for polygenic effects. Basic multicellular organisms such as *Dictyostelium* provide a link between cell culture and vertebrate models, however some caution should be taken due to the low level of identity between the *Dictyostelium* and vertebrate CLN5 primary sequences. The use of brain-derived cells is important in studying the neural basis of Batten disease as there is increasing evidence for neural specific roles of several Batten associated genes including CLN1, 3 and 6 [150–154]. The achievement of a structure for human CLN5 (PDB 6R99) will allow comparative modelling and predictions of possible catalytic activity and effects of mutations on structural integrity. If CLN5 is indeed a lysosomal protease it will be important to characterise its substrates in neurons, as these could be targets for interventions.

While gene therapy can be achieved with limited knowledge of the protein defects and normal protein function, natural histories of both human cases and model systems are critical to developing therapies and testing efficacy. However, gene therapy has limitations – no vector has 100% transduction efficiency and careful consideration needs to be given to regulation of expression and cell type-specific targeting. Further studies on the regulation of transcript abundance, translation and post-translational modification at the cell-type and tissue level are still required in order to fully optimise gene therapy. In addition, although CLN5 can be secreted from transduced cells, its essential role in glial cells in the brain has not been established. Most gene therapy vectors in common clinical use target neurons: a requirement to target glial or other non-neuronal cells in the brain will require development of new vectors. In addition, as gene therapy improves brain function and survival, the integrity and function of other organs may be compromised. Already there is an obvious need to target the retina in CLN5. Other organs are yet to be studied. Both CLN5 mouse and sheep models provide strong face validity for aspects of disease progression and sheep have already proved useful in development of a gene therapy strategy for CLN5 Batten disease. Sheep and mouse studies have also provided strong natural history landmarks including pathological, imaging and behavioural measures. Both these models can easily be used to test efficacy of therapies given at different stages of disease and with different routes of administration. While sheep have numerous advantages including a more human-like brain structure and size as compared to rodents, they are restricted in terms of research costs (most studies are completed on only 3–4 sheep per group). In addition, any oral drug delivery is complicated by their ruminant anatomy. For this reason, some researchers have turned to transgenic pig models (J. Weimer, Sanford Research, SD, personal communication).

While structure underpins function, an understanding of basic cellular biology and how mutations cause complex changes in neuronal function and neurodegeneration remains to be determined for all neurodegenerative diseases. Understanding CLN5 function not only has the potential for treatments of CLN5 Batten disease. CLN5 allelic variation in Alzheimer’s disease and the common pathologies of lysosome dysfunction in many other neurodegenerative diseases mean that future focus on CLN5 will likely drive our understanding of brain function more widely.

Families are a critical component of research. Without their support and insight, many key symptoms and hints towards function of NCL proteins would be missed. Furthermore, families and foundations are often the drivers of integration and collaborations between researchers, clinicians and biotechnology companies. Therefore, a combined effort between the families, foundations and researchers and clinicians will drive strategies towards finding a cure for CLN5 Batten disease.
