# Correction: A lysosomal enigma CLN5 and its significance in understanding neuronal ceroid lipofuscinosis

**DOI:** 10.1007/s00018-023-05047-5

**Published:** 2024-01-18

**Authors:** I. Basak, H. E. Wicky, K. O. McDonald, J. B. Xu, J. E. Palmer, H. L. Best, S. Lefrancois, S. Y. Lee, L. Schoderboeck, S. M. Hughes

**Affiliations:** 1 Neurodegenerative and Lysosomal Disease Laboratory, Department of Biochemistry, School of Biomedical Sciences, Brain Health Research Centre, University of Otago, 710 Cumberland Street, Dunedin, 9016 New Zealand; 2 School of Biosciences, Cardiff University, Sir Martin Evans Building, Museum Avenue, CF10 3AX Wales, UK; 3 Centre INRS-Institut Armand-Frappier, INRS, H7V 1B7 Laval, Canada; 4 Department of Anatomy and Cell Biology, McGill University, H3A 2B2 Montreal, Canada; 5 Department of Biochemistry and Molecular Biology, University of Kansas Medical Center, 66160 Kansas City, KS USA

**Correction: Cellular and Molecular Life Sciences 78, 4735–4763 (2019)**

10.1007/s00018-021-03813-x.

In this article ref. ‘Leubben et. al’ was incorrect and should have been ‘Luebben et. al.’.

The original article has been corrected.

